# Preclinical and first-in-human safety studies on a novel magnetism-based haemofiltration method

**DOI:** 10.1038/s41598-024-64379-9

**Published:** 2024-06-18

**Authors:** Nicole Waalders, Dirk van Lier, Jelle Gerretsen, Lucy Moran, Kerstin A. Stegmann, Will Twigger, Cristina Blanco-Andujar, George Frodsham, Matthijs Kox, Peter Pickkers

**Affiliations:** 1 Department of Intensive Care Medicine, Radboud University Medical Center, Nijmegen, The Netherlands; 2 Radboud University Medical Center, Radboud Center for Infectious Diseases (RCI), Nijmegen, The Netherlands; 3 MediSieve Limited, London, UK

**Keywords:** Sepsis, Preclinical research

## Abstract

Extracorporeal haemofiltration devices that selectively remove cytokines could represent an adjunctive treatment in inflammatory diseases. One such device is the “IL-6-Sieve”, wherein magnetic Anti-IL-6 Beads are introduced into an extracorporeal circuit via a Bead Adapter and then removed along with any surface-bound interleukin (IL)-6 by a Filter deployed in a Magnet, before the blood is returned to the patient. We report here on a series of animal studies, and a first-in-human study, on the safety of the IL-6-Sieve. Evaluations focused on the: (a) safety of Filter and Magnet placed in an extracorporeal circuit in sheep; (b) safety of Anti-IL-6 Beads—directly infused intravenously as worst case scenario of misuse; or injected into an extracorporeal circuit using the Bead Adapter, Filter, and Magnet as intended—in sheep; (c) biodistribution of Anti-IL-6 Beads intravenously infused in mice; and (d) safety of Filter and Magnet placed in an extracorporeal circuit in healthy volunteers. No serious adverse events or significant changes in vital signs or routine laboratory parameters occurred in any of the animals or humans. Although safety of the IL-6-Sieve requires further study, these initial evaluations represent a promising start for the translation of this new blood purification modality into clinical use.

## Introduction

Acute inflammatory diseases represent an enormous healthcare problem. Sepsis, defined as life-threatening organ dysfunction caused by a dysregulated host response to infection, is the most prevalent severe inflammatory disease, accounting for approximately 20% of all deaths worldwide. This makes it the leading cause of death in the intensive care unit (ICU) and the leading cause of death worldwide^1^. The dysregulated immune response to infection in sepsis, varying from a pronounced inflammatory state (i.e., hyperinflammation) to an immunosuppressive state (i.e., immunoparalysis), is complex and has been intensively studied over the past decades^2,3^. The hyperinflammatory phase is characterized by elevated concentrations of pro-inflammatory cytokines, such as interleukin (IL)-6. In critically ill patients with COVID-19 or cytokine release syndrome (CRS) caused by CAR-T cell therapy, IL-6 has been shown to play a crucial pathogenic role, as blocking the IL-6 receptor improves clinical outcome in these conditions^4–6^. For (bacterial) sepsis, several immunosuppressive drugs have been tested^7^. However, as none of these pharmacological therapies were shown to improve outcome in comparative trials, standard sepsis care is currently still focussed on the timely administration of antibiotics, source control, and organ supportive therapies^7,8^.

Extracorporeal blood purification therapies—including haemofiltration, haemoperfusion, and plasma filtration—have also been investigated as adjunctive therapies to modulate the immune response in patients with sepsis^9–11^. Blood purification is based on the concept that excessive concentrations of circulating inflammatory mediators, such as cytokines or bacterial endotoxin play an important role in the pathogenesis of sepsis^12–14^. Removing these mediators may limit organ damage and could consequently improve clinical outcome in septic patients^13,15^. Currently, several haemoperfusion devices based on adsorption are commercially available and used in clinical practice. These include Toraymyxin (Toray Industries, Japan), the CytoSorb® cartridge (Cytosorbents Inc, USA), the Jafron HA cartridge (Jafron Biomedical, China), and the oXiris filter (Baxter, USA). Toraymyxin, a polymyxin B based column, specifically targets endotoxins, whereas the other adsorbers use a non-specific adsorption approach to remove cytokines. Although in vitro studies as well as experimental animal and human studies have shown effective removal of endotoxin or cytokines by haemoadsorption, it remains controversial whether these effects translate to better outcomes in sepsis patients^16–21^.

The principle behind most haemoadsorption devices is the binding of substances based on their molecular size. A major drawback of this non-specific binding approach is that it also leads to the removal of potentially beneficial inflammatory mediators as well as essential drugs, e.g. antibiotics^10^. Therefore, more selective removal of inflammatory mediators is desirable, and novel haemofiltration techniques based on the use of high-gradient magnetic separation in conjunction with antibodies have been proposed to achieve this^22^. These techniques are similar to methods widely used in laboratories where functionalised magnetic particles bind to specific cell types in a heterogenic mixture after which the cells of interest are isolated by magnetic separation^23^. In the setting of haemofiltration, exposure of blood to magnetic beads coupled to a (bio)molecule that specifically binds the intended target followed by magnetic separation can selectively remove components from the circulation^22,24^.

Based on this principle, a novel haemofiltration device called the “IL-6-Sieve” has been developed by MediSieve Limited (UK). The IL-6-Sieve comprises four component devices—Filter, Magnet, Bead Adapter, and Anti-IL-6 Beads (all of which are manufactured by MediSieve Limited (UK))—which are used in conjunction with a user-supplied extracorporeal circuit, blood pump, and vascular access device. Briefly, the principle of operation is that the Anti-IL-6 Beads are continuously infused into an extracorporeal circuit via the Bead Adaptor while the patient’s blood is circulating through the extracorporeal circuit, and then removed—using the Filter and Magnet—before the blood is returned to the patient. In this way IL-6 can be removed from the circulating blood, reducing IL-6-driven hyperinflammation.

The safety of the IL-6-Sieve needs to be fully assessed before (randomised controlled) trials in septic patients can be conducted to evaluate the clinical efficacy of this novel system. In the present work, we report on the safety of the extracorporeal use of the Filter and Magnet, with and without infusion of the Anti-IL-6 Beads into the circuit via the Bead Adapter, in sheep. Furthermore, direct intravenous injection of Anti-IL-6 Beads was used to assess safety in sheep and biodistribution in mice. Of note, this represents the worst case scenario of misuse and not the intended clinical use. Finally, we present the results of a first-in-human safety study of the deployment of the Filter and Magnet in an extracorporeal circuit.

## Materials and methods

**Figure 1:**
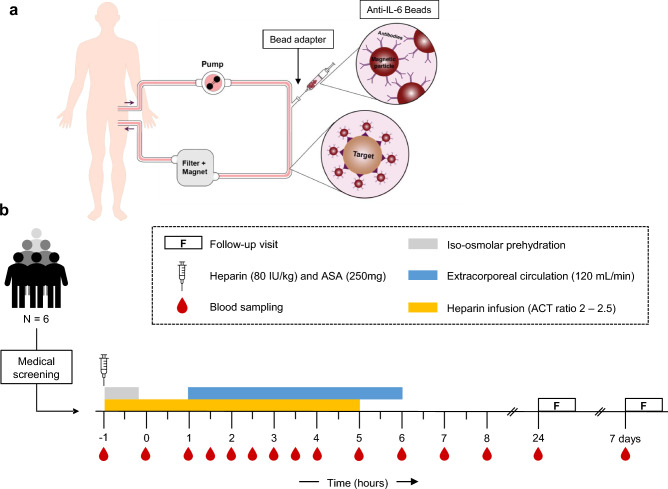
Overview of the setup of the IL-6-Sieve and the first-in-human study. (**a**) Schematic overview of the intended use of the IL-6-Sieve. Note that for the first-in-human study, no Anti-IL-6 Beads were infused. (**b**) Schematic overview of the first-in-human study. ASA, acetylsalicylic acid; ACT, activated clotting time**.**

The mechanism of action of the IL-6-Sieve is described in detail in the Supplementary Methods. In short, when the Filter is placed inside the Magnet, the magnetic field allows for magnetic components (such as Anti-IL-6 Beads) to be retained within the Filter. The Bead Adapter is placed in the extracorporeal circuit before the filter to allow for the infusion of magnetic Beads via a syringe pump. An overview of the setup of the intended use of the IL-6-Sieve is shown in Fig. 1a.

### Animal studies

All animal experiments were approved by the appropriate animal ethics committees, performed according to local applicable guidelines and regulations (see Supplementary Methods), and reported according to the ARRIVE guidelines. In the animal experiments, no blinding or randomisation was applied.

### Safety of the filter and magnet

#### Study procedures

To assess the safety of the Filter and Magnet component devices, without the infusion of the Anti-IL-6 Beads and without employing the Bead Adapter, a study in three healthy female sheep (number 1–3, Mule breed, Park Farm, UK) was performed at the Northwick Park Institute for Medical Research (Harrow, UK) in compliance with Good Laboratory Practice (GLP) regulations. Animals completed an acclimatisation period of two weeks and were kept group-housed until 2 days prior to the surgery, after which they were single housed under temperature and humidity control with a 12 h/12 h light–dark cycle. Animals were weighed on arrival, on the day of surgery and the weekly up until day 28 (see Supplementary Table S1 for an overview of the study procedures and measurement timepoints). Hay and Champion Ewe 18 Nuts was provided daily and water was available ad libitum. The animals were fasted approximately 16 h prior to surgery and drinking water was replaced with Lectade Plus rehydration solution.

The sheep were anesthetized intravenously via a cannula in the cephalic vein prior to endotracheal intubation after which anaesthesia was maintained using isoflurane. Two 14G cannula were inserted, one in the right jugular vein towards the brain and the other one in the left jugular vein towards the heart. An intravenous bolus of 3000 units (IE) of unfractionated heparin was administered to the sheep after which a continuous infusion of 750 IE/h was initiated on the Aquarius Haemofiltration pump (Nikkiso Aquarius platform, Nikkiso Europe GmbH, Germany). Priming of the Filter and the circuit (Haemotronic S.p.A., Italy) was performed with 0.9% saline. The Filter was then deployed within a Magnet and extracorporeal circulation in order to establish the capability within the Filter, and treatment was performed for two consecutive hours at a flow rate of 100 mL/min. Blood samples were obtained at defined timepoints during the treatment procedure and follow-up period. After treatment was completed, blood was returned to the sheep and the wounds were sutured, after which the sheep recovered in the animal accommodation area. Post-surgery analgesia consisted of an intramuscular bolus of 0.01 mg/kg buprenorphine twice daily for three days, as well as a single subcutaneous bolus of 2 mg/kg carprofen daily for three days. Daily observations were performed, and the sutures were removed on day 14. After the last blood withdrawal on day 28, the sheep were euthanised under deep anaesthesia using pentobarbitone.

#### Sample collection and measurements

An overview of the samples obtained on the different timepoints is provided in Supplementary Table S1. Haematology samples were immediately analysed using the ADVIA 2120 Analyser (Siemens Healthineers, Germany). Furthermore, osmotic fragility of whole blood samples was immediately analysed using a spectrophotometer. Serum samples for clinical chemistry were stored at – 20 °C before analysis on the IL650 analyser (Werfen, UK).

Samples of multiple organs and bone marrow were obtained post-mortem for histology and cell morphology analysis. Histology samples of the lung, liver, heart, kidney, spleen, pancreas, large and small bowel, adrenal glands, and a lymph node were stored in 10% neutral buffered formalin (NBF) for a minimum of 48 h. Samples were then embedded in paraffin wax after which they were stained with haematoxylin and eosin and examined for abnormalities. Blood smears and bone marrow smears were stained with a May Grunwald-Giesma stain, after which they were analysed by the pathologist for cell morphology by transmitted light microscopy.

### Safety of the Anti-IL-6 Beads and Bead Adapter, and of the IL-6-Sieve

#### Study procedures

The safety of the Anti-IL-6 Beads, either directly infused; or used in conjunction with the Bead Adapter; or used in conjunction with the Bead Adapter, Filter and Magnet according to the intended clinical use (i.e., as the IL-6-Sieve device); was assessed in two different sheep experiments performed at the Royal Veterinary College (Hatfield, UK) using a total of two female Mule cross sheep aged 3–5 years. Sheep had at least one week of acclimatisation prior to the start of the study. The sheep were group-housed and moved to an individual pen prior to the procedure with natural temperature, light and ventilation. Water and food were available ad libitum, supplemented with a restricted amount of commercial pelleted ewe diet twice daily based on their minimum basal and metabolic energy requirements. The sheep were fasted 12–18 h prior to the experiment. For a detailed overview of the anaesthesia, cannulation, and monitoring procedures, see Supplementary Methods.

In the first experiment, Anti-IL-6 Beads were intravenously injected in one awake sheep (number 4) to assess adverse events in the case that the Anti-IL-6 Beads might accidently enter the circulation. Of note, this is different from the intended clinical use and seen as worst case scenario in case of misuse. As no extracorporeal circulation was performed in this experiment, the sheep was not connected to an extracorporeal circuit and no heparin was administered. Three 10 mL vials containing Anti-IL-6 Beads (10 mg/mL) were diluted each to a volume of 50 mL using 0.9% saline, which was subsequently intravenously injected in approximately 15 min. Further details on the Anti-IL-6 Beads are not provided as it is part of Intellectual Property of MediSieve Limited. The sheep was euthanised 4 h after injection of Anti-IL-6 beads by intravenous administration of 20 mL Somulose Solution.

In the second experiment, one anaesthetised sheep (number 5) underwent haemofiltration using the Aquarius Haemofiltration pump, with infusion of Anti-IL-6 Beads via the Bead Adapter connection into the extracorporeal circuit containing a Filter and Magnet for four consecutive hours, the intended length of the IL-6-Sieve treatment. A similar anticoagulation protocol, as described for the first experiment was used; i.e. a bolus of 50 IE/kg unfractionated heparin followed by continuous infusion of 10 IE/kg/h adjusted to maintain an ACT > 200 s. Three 10 mL vials containing Anti-IL-6 Beads (10 mg/mL) were diluted each to a volume of 50 mL using 0.9% saline, which was subsequently intravenously infused 5 min after initiation of the IL-6-Sieve treatment at a flow rate of 0.6 mL/min. After the IL-6-Sieve treatment was finished, the sheep was euthanized by injection of pentobarbital without recovery from anaesthesia. The Filter including the beads was disposed of. Investigation into Bead escape was previously carried out on benchtop experiments using beads labelled with a fluorescent tag. Analysis of the solution exiting the filter revealed levels below the limit of detection.

#### Sample collection and measurements

For the first experiment, blood samples were obtained prior to the Anti-IL-6 Beads injection and 30, 120 and 240 min after of Anti-IL-6 Beads injection. Blood samples in the second experiment, when the sheep was connected to the extracorporeal circuit, were obtained on different timepoints (see Supplementary Table S2). Haematology, clinical biochemistry and IL-6 concentration were measured on all timepoints, whereas total iron was only measured prior to the treatment and after 240 min. Details on sample measurements are provided in the Supplementary Methods.

### Biodistribution of the Anti-IL-6 Bead﻿s

#### Study procedures

In this study, the biodistribution of Anti-IL-6 Beads was assessed after direct intravenous injection in nine adult male C57BL/6 J mice using T2*- and T2-weighted magnetic resonance imaging (MRI) at Charles River Discovery Services Finland (Kuopio, Finland). Details on exclusion criteria, housing, and habituation are described in the Supplementary Methods. This study was designed to get insight into which organ Anti-IL-6 Beads would accumulate in the case of direct intravenous administration, which would be the worst case scenario of misuse, their clearance from the system and which clinical parameter need to be focused on specifically in future first-in-human studies using Anti-IL-6 Beads.

Mice were assigned to three groups (n = 3 per group) in which imaging of the abdominal organs was performed at baseline (all groups) and at 30 min (group 1), 2 h (group 2) or 24 h (group 3) after an intravenous bolus injection of 100 µL Anti-IL-6 Beads (concentration 1 mg/mL in 0.9% saline) in the tail vein. This dose was chosen after establishing the detection limit of Anti-IL-6 Beads by MRI. The area of interest for the abdominal organs contained liver, kidneys and spleen. Imaging of the brain was only performed on mice in group 3 at 25 h post-bead injection. Prior to MRI imaging, mice were anesthetised with 5% isoflurane. During imaging, the isoflurane concentration was reduced to 1.5%-2.0% and body temperature was maintained at 36.5 ± 1.5 °C with a homeothermic blanket system. Mice in group 1 were kept under anaesthesia in between baseline and 30 min scans, whereas the baseline scan of mice in the other groups were done beforehand and the mice were awake before their final scan. To perform MRI, mice were positioned in the magnet bore in a standard orientation relative to the gradient coils. For details on the MRI procedure, see Supplementary Methods. Following the last MRI scan, body weight was recorded, and mice were euthanised by intraperitoneal injection with 180 mg/kg pentobarbital.

### First-in-human study

#### Study design and ethics

This single-centre, non-randomised, prospective phase-1 study assessed the safety of the Filter and Magnet component devices only; i.e. without the Anti-IL-6 Beads infusion and without the use of the Bead Adapter. It was approved by the local ethics committee (METC Oost-Nederland; reference nos. NL80026.000.21 and CMO 2022-13606) and registered at ClinicalTrials.gov (NCT05713188, 06/02/2023). All study procedures were performed in accordance with the declaration of Helsinki and its most recent revisions.

#### Study population

Six healthy male and female volunteers aged 18–50 years were recruited. All subjects provided written informed consent and were included after medical screening, which consisted of medical history, physical examination, a 12-lead electrocardiogram and routine laboratory tests. Exclusion criteria were smoking, pregnancy or lactating, a body mass index under 18 kg/m^2^ or above 30 kg/m^2^, use of any medication, allergies to any of the investigational products or signs of acute illness within two weeks prior to the start of the study.

#### Experimental procedures

An overview of the study procedures is displayed in Fig. 1b. Participants refrained from alcohol and caffeine (24 h) as well as from food and drink consumption (12 h) prior to the start of the experimental day. On this day, subjects were admitted to the research unit of the Intensive Care department of the Radboud university medical center (Radboudumc, the Netherlands) for approximately 11 h. An arterial catheter was placed in the radial artery (BD Infusion Therapy Systems, USA) for continuous blood pressure monitoring and frequent blood withdrawal. Furthermore, a venous cannula was placed in the antebrachial vein to administer (pre)hydration fluids and medication. A haemodialysis catheter (High Flow Double Lumen 13Fr 250 mm, Joline, Germany) was subsequently placed in the right femoral vein under local anaesthesia and ultrasound guidance. Heart rate and intra-arterial blood pressure were measured using a 3-lead electrocardiogram (M50 Monitor, Philips, the Netherlands) and a radial artery pressure transducer (Edwards Lifesciences, USA), respectively. To keep the protocol in line with a possible future experimental endotoxemia study (in which bacterial lipopolysaccharide is administered to elicit a controlled systemic inflammatory response), subjects were prehydrated with 1.5 L of sodium chloride 0.45%/glucose 2.5% administered in one hour. This is the standard procedure for endotoxemia studies previously used to prevent vasovagal responses^25^. Subjects were subsequently hydrated by infusing the same fluids at a rate of 150 mL/h for 8 h. Blood samples were serially obtained for laboratory safety and immunological tests. Body temperature was measured every 30 min using a tympanic thermometer (FirstTemp Genius 2, Covidien, Ireland). Flu-like symptoms and overall illness were scored every 30 min using a numeric six-point Likert scale (0 = no symptoms, 5 = worst ever experienced) and an eleven-point scale (0 = no malaise, 10 = worst ever experienced) respectively.

For each experiment, a Filter connected to an extracorporeal circuit was deployed into a Magnet mounted on a haemofiltration machine with a dedicated line set (Nikkiso Aquarius platform and Aqualine adult tube set, Nikkiso Europe GmbH, Germany). Prior to connection to the subject, the circuit and the Filter were primed with 5000 IE of unfractionated heparin per litre of NaCl 0.9% according to the manufacturer’s instructions. Initially, an intravenous bolus of unfractionated heparin of 80 IE/kg with a continuous infusion of 20 IE/kg/hour for six hours was used for anticoagulation. However, due to clot formation in the filter itself, these experiments were terminated prematurely. To resolve this, an intravenous bolus of acetylsalicylic acid (ASA) of 250 mg was administered together with the heparin bolus and infusion. The ACT was measured every 30–60 min with a point-of-care analyser (Hemochron Signature Elite, UK) and heparin was adjusted accordingly to keep the ACT-ratio between 2- and 2.5-times baseline ACT. Extracorporeal circulation was started two hours after administration of anticoagulants and subjects were treated for five consecutive hours with a constant flow rate of 120 mL/min. At the end of the treatment, blood was returned to the subject, and they were disconnected from the extracorporeal circuit. Subjects remained at the research unit for observation for two more hours before removal of the catheters and discharge.

Subjects attended follow-up visits 24 h and 7 days after the experiment day. Blood samples were obtained, and the cannulation sites were inspected. Adverse events (AEs) were recorded during the experiment day and both follow-up visits. An AE was defined as any untoward medical occurrence, unintended disease or injury, or untoward clinical signs (including abnormal laboratory findings) in subjects, whether or not related to the Filter and Magnet. All AEs were assessed by the investigator with regard to severity (mild, moderate, or severe) and their relation to the study protocol and/or the Filter and Magnet (unrelated, possibly, probably or causally).

#### Sample collection and measurements

Laboratory safety tests, including hemocytometry, electrolytes, coagulation, liver and kidney function, and metal analysis were performed on citrate, lithium-heparin or EDTA-anticoagulated containers by the Department of Laboratory Medicine of Radboudumc using routine analysis methods also used for clinical samples. Reference ranges were obtained from this department. Blood for cytokine measurements was drawn in EDTA-anticoagulated vacutainers and centrifuged directly after withdrawal (2000 g for 10 min at 4 °C), after which plasma was stored at -80°C until analysis. Concentrations of several cytokines, including tumour necrosis factor (TNF), IL-1 receptor antagonist (RA), IL-6, IL-8, IL-10, macrophage inflammatory protein (MIP)-1α, MIP-1ß, monocyte chemoattractant protein (MCP)-1, granulocyte colony-stimulating factor (G-CSF) and interferon-γ-induced protein (IP)-10 were determined batchwise using a simultaneous Luminex assay (Milliplex, Millipore, USA) according to the manufacturer’s instructions. The lower limit of detection was 3.2 pg/mL for all measured cytokines.

### Statistical analysis

There was no formal power calculation in the animal experiments due to the nature of the studies (safety and tolerability). For the *safety of the Filter and Magnet* and the *Safety of the anti-IL-6 Beads and Bead Adapter, and of the IL-6-Sieve* studies, data are displayed per individual animal. For the *biodistribution of the Anti-IL-6 Beads* study, relative signal changes in T2*-weighted gradient echo and T2-weighted spin echo are represented as mean ± standard deviation (SD). In every region of interest, different timepoints were compared using one sample t-tests against zero followed by Bonferroni correction. A p-value of < 0.05 was considered statistically significant. For MRI imaging, manual regions of interest for volumes were determined using in-house written Matlab software (MathWorks Inc., USA). For the *first-in-human* study, a formal power calculation was not performed due to the nature of the study. A sample size of six subjects is common for a first-in-human study and provides sufficient data to assess the safety of the IL-6-Sieve^26^. Demographic data are displayed as median with range, whereas vital signs, and laboratory and immunological safety data are represented per individual subject and as median with interquartile range. A total symptom score was calculated as the sum of all symptom scores, including the overall illness score. Data analysis and visualisation were performed using R version 4.1.3 (The R Foundation for Statistical Computing, Vienna, Austria).

## Results

### Animal studies

#### Safety of the Filter and Magnet

Extracorporeal circulation for 2 h did not cause AEs or mortality in the three sheep included. There were also no clinical signs of illness related to the treatment. All animals initially lost some weight post-surgery, but they started to put on weight after day 14 (see Supplementary Table S3).

**Figure 2:**
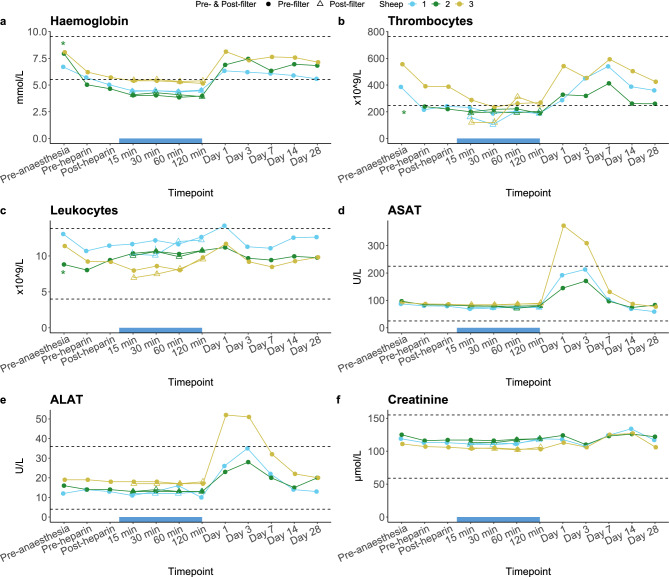
Changes over time in laboratory parameters in the GLP safety study on the IL-6-Sieve’s Filter and Magnet component devices in sheep. Data are displayed per individual animal (coloured lines). The extracorporeal circulation period is indicated by the blue bar on the x-axis. During this period, circles represent the “Pre-filter” values (sampled at the circuit-to-Filter entry point), whereas the triangles represent the “Post-filter” values (sampled at the Filter-to-circuit exit point). Dashed horizontal lines indicate the reference values. * = Sheep number 2 had a clotted blood sample on timepoint ‘Pre-anaesthesia’, therefore this timepoint was excluded for thrombocytes but not for haemoglobin and leukocytes although it could have influenced the analysis. ASAT, aspartate aminotransferase; ALAT, alanine aminotransferase.

A decrease of approximately 20% in haemoglobin and 40% in thrombocytes was observed after initiation of anaesthesia, but before administration of heparin, indicating this initial drop is caused by drugs used for anaesthesia (Fig. 2a, b respectively). Haemoglobin subsequently slightly decreased further up until 15 min into the treatment, after which it remained stable and increased to approximately baseline concentrations post-treatment (Fig. 2a). From the post-heparin timepoint up until 30 min into treatment, an additional small decrease in thrombocytes was observed, after which numbers remained stable during treatment. On day 1 after treatment, thrombocytes were back to pre-anaesthesia levels. Post-filter thrombocyte counts, sampled at the Filter-to-circuit exit point, were lower than pre-filter counts, sampled at the circuit-to-Filter entry point, at 15 and 30 min into treatment, whereas they were similar at 60 and 120 min. Leukocyte numbers slightly decreased between baseline and the pre-heparin timepoint, after which they remained stable throughout treatment (Fig. 2c). For both aspartate aminotransferase (ASAT) and alanine aminotransferase (ALAT), an increase above the upper limit of normal was observed 1 and 3 days following treatment, after which concentrations reverted to normal (Fig. 2d and erespectively). No relevant changes were observed for creatinine concentrations at any of the timepoints (Fig. 2f). Creatinine kinase (CK) concentrations gradually increased to approximately two-fold above the upper limit of normal until 3 days post-treatment, after which they normalised (Supplementary Table S4). Other laboratory parameters showed no relevant changes over time (Supplementary Table S4).

There were no substantial differences in the mean corpuscular fragility of red blood cells during the experimental procedure and the follow-up period (range 5.9–6.8 g NaCl/L; normal range is 6.0–6.5 g NaCl/L in fresh blood samples). Furthermore, blood smears showed no morphological abnormalities for all but one sheep, in which slight crenation (irregular undulation of the red blood cell outer membrane) of the red blood cells from timepoint pre-anaesthesia until 15 min into treatment was observed.

Macroscopically, all organs appeared normal, although one sheep had small lumps on a lung lobe. Microscopic analysis revealed that this was due to pre-existing fibrosis and mineralisation secondary to pre-existing lung infection, which was also observed, albeit to a more limited degree, in the other sheep. Also, small deposits of live lungworm (Dictyocaulus Filaria) were observed, which is not uncommon in British organically farmed sheep. Furthermore, in all animals some sarcocystis was observed within normal myocardium, which is an endemic parasitic infection common in British sheep. In the other organs, no abnormalities were observed upon microscopic analysis.

#### Safety of the Anti-IL-6 Beads and Bead Adapter, and of the IL-6-Sieve

**Figure 3:**
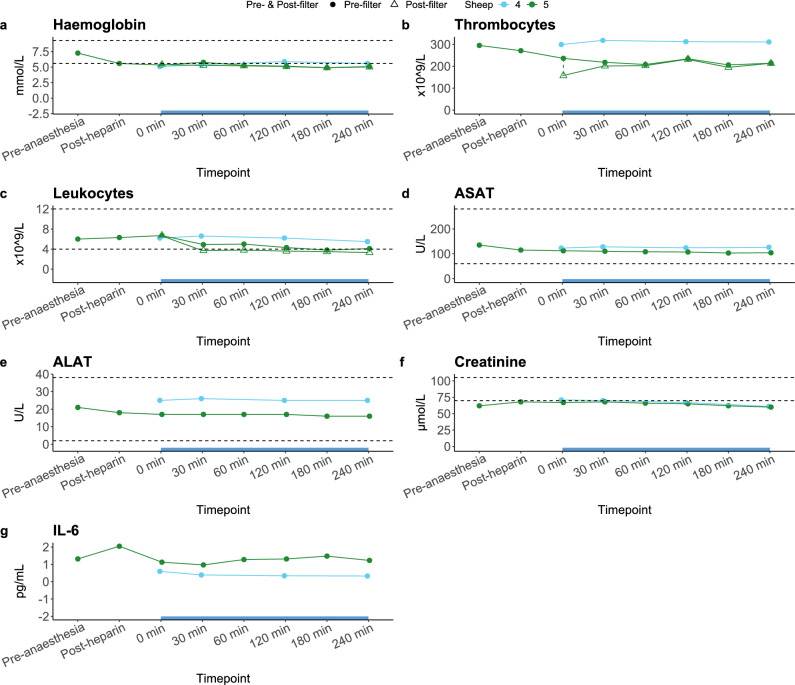
Changes over time in laboratory parameters in the safety study on the IL-6-Sieve’s Anti-IL-6 Beads, Bead Adapter, Filter, and Magnet component devices in sheep. Data are displayed per individual animal (coloured lines). Sheep 4 = direct injection of Anti-IL-6 Beads into the circulation without extracorporeal circulation or administration of anaesthesia and heparin; Sheep 5 = haemofiltration and infusion of Anti-IL-6 Beads into the extracorporeal circulation system via the Bead Adapter. The extracorporeal circulation/haemofiltration period is indicated by the blue bar on the x-axis (only applicable to sheep 5). During this period, circles represent the “Pre-filter” values (sampled at the circuit-to-Filter entry point), whereas the triangles represent the “Post-filter” values (sampled at the Filter-to-circuit exit point). Dashed horizontal lines indicate the reference values. ASAT, aspartate aminotransferase; ALAT, alanine aminotransferase.

In the first experiment (sheep 4), Anti-IL-6 Beads were intravenously administered within 15 min to one awake female sheep with a bodyweight of 106.5 kg. No extracorporeal circulation was performed. Throughout the treatment and 4-h observation period, the sheep was alert and responsive with a normal body rectal temperature (data not shown). A mild increase in heart rate and respiratory rate was observed halfway into the bead injection but normalised 5 min after all Anti-IL-6 Beads had been injected. There were no relevant changes in haemoglobin, thrombocytes or leukocytes at any of the timepoints (Fig. 3a–c respectively). Total iron and calcium concentrations were below the normal range at baseline (prior to Anti-IL-6 Bead injection; i.e. timepoint 0 min) and at the end of the observation period. LDH was elevated at all timepoints with no changes in levels between timepoints (Supplementary Table S5), whereas ASAT, ALAT and creatinine were within normal or just under the normal range at all timepoints with only minor changes in between (Fig. 3d,f respectively). Plasma concentrations of IL-6 showed no relevant changes over time (Fig. 3g).

In the second experiment, one female sheep (sheep 5) with a bodyweight of 99 kg underwent IL-6-Sieve haemofiltration with infusion of Anti-IL-6 Beads via the Bead Adapter into an extracorporeal circuit containing a Filter and Magnet for four consecutive hours. This is the intended clinical use and the intended clinical period of treatment of the complete IL-6-Sieve device. A mild decrease in haemoglobin and thrombocytes was observed from timepoint pre-anaesthesia until the post-heparin timepoint due to administration of intravenous fluid (Fig. 3a–c respectively). Haemoglobin remained stable during the treatment, whereas thrombocytes slightly decreased until 60 min following the start of treatment, after which they remained stable. Leukocytes decreased 30 min following the start of the treatment to levels just above the normal range at the end of treatment. ASAT, ALAT and creatinine showed a slight downward trend over time but stayed within normal range, except for creatinine which was slightly below normal range at the end of treatment (Fig. 3d, f). Total iron concentrations were below the normal range at baseline and at the end of the treatment, calcium concentrations were below the normal range at all timepoints, and LDH was above the normal range at all timepoints, with the highest being the pre-anaesthesia timepoint (Supplementary Table S5). IL-6 concentrations (Fig. 3g) showed no relevant changes over time.

The results of other laboratory parameters are listed in Supplementary Table S5 for bothexperiments.

#### Biodistribution of the Anti-IL-6 Beads

In this study, a bolus of Anti-IL-6 Beads was administered intravenously to nine mice; and abdominal organs were imaged by MRI at baseline and at 30 min (group 1, n = 3), 2 h (group 2, n = 3) or 24 h (group 3, n = 3) after injection. The brain was only imaged by MRI in group 3. No haemofiltration was performed in these mice. Ex vivo imaging of a range of concentration was performed (data not shown) to establish limit of detection and select a dose to be administrated which will allow for a signal to seen by MRI. T2-weighted spin echo and T2*-weighted gradient echo data of one animal in group 1 was excluded due to image artefacts and low signal to noise ratio. Body weight of the mice was 24.2 ± 0.8 g.

**Figure 4:**
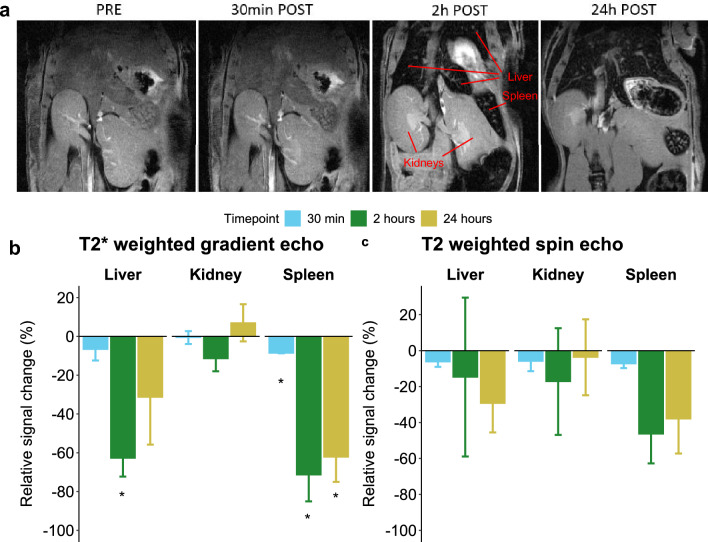
Contrast change in abdominal organs of mice infused with magnetic Beads in the biodistribution of Anti-IL-6 Beads study. (**a**) Dynamics of the contrast change in T2* weighted gradient echo images at baseline, 30 min, 2 h and 24 h after Anti-IL-6 Bead injection. MRI voxel size (spatial resolution) of 100 × 100 × 500 µm^3^. (**b**) Relative signal drop as compared to baseline images in T2* weighted gradient echo images in liver, kidney and spleen 30 min (n = 2), 2 h and 24 h (both n = 3) after Anti-IL-6 Beads injection. Data are shown as mean ± standard deviation. P-values were computed by one sample t-test for every individual organ and timepoint against zero followed by Bonferroni correction. * = p < 0.05. (**c**) Relative signal drop as compared to baseline images in T2 weighted spin echo images in liver, kidney and spleen 30 min (n = 2), 2 h and 24 h (both n = 3) after Anti-IL-6 Beads injection. Data are shown as mean ± standard deviation. P-values were computed by one sample t-test for every individual organ and timepoint against zero followed by Bonferroni correction.

The abdominal T2*-weighted and T2-weighted MRI revealed significant drops in signal in the liver and spleen, but not in the kidneys at 2 and 24 h after administration of the Anti-IL-6 Beads, which is indicative of the presence of the magnetic beads (Fig. 4a). T2*-weighted gradient echo showed a similar pattern as found by MRI. Signal intensity changes in T2*-weighted gradient echo and T2-weighted spin echo were quantified to confirm the visual changes in the images (Fig. 4b, c respectively). A significant relative signal intensity drop was observed in T2*-weighted gradient echo at 30 min post-administration compared to baseline in the spleen (− 8.6% ± 0.1, p < 0.05), at 2 h post-administration compared to baseline in the liver (− 62.8% ± 5.5; p < 0.05) and the spleen (− 71.4% ± 7.9; p < 0.05), and for the spleen also at 24 h post-administration compared to baseline (− 62.2% ± 7.4; p < 0.05). In the liver, a relative signal drop at 24 h post-administration was also observed, however this was less than at 2 h post-administration and not statistically significant compared to baseline (− 31.4% ± 14.1; p = 0.47). In the kidney, the relative signal intensity changes were small and not statistically significant at any of the timepoints. For T2-weighted spin echo, the observed changes were not statistically significant in any organ at any of the timepoints compared to baseline, although similar to the T2*-weighted gradient echo data, the largest signal drops were observed in the spleen.

Brain contrast in high resolution T2*-weighted 3D gradient echo and 3D MGE images showed no significant changes in the brain parenchyma and cortex 25 h post-Anti-IL-6 Beads administration compared to baseline (relative signal intensity change normalised to muscle of 3.3 ± 2.6% for parenchyma and 11.6 ± 3.3% for cortex, respectively) (Supplementary Fig. S1). All suspect radiological findings that matched the beads based on their MRI properties were inspected at 3D 100 × 100 × 100 µm^3^ spatial resolution T2*-weighted and multi echo images. These findings appeared to be adjacent or associated with vascular structure, therefore indicating no accumulation of Anti-IL-6 Beads in the brain. Further, no other brain abnormalities (such as ischemic lesions) were detected.

### First-in-human study

Six healthy male and female volunteers (ratio 1:1) were included in this study in which the safety of extracorporeal circulation using the Filter and Magnet component devices (without the infusion of Anti-IL-6 Beads or the use of the Bead Adapter) was tested for five consecutive hours (i.e., one hour beyond the intended clinical use of the IL-6-Sieve device). Baseline demographic characteristics obtained during the screening visit are presented in Table 1.

**Table 1:** Demographic characteristics of the healthy volunteers enrolled in the first-in-human study.

	Total (n = 6)	Men (n = 3)	Women (n = 3)
Age (years)	24 (19–25)	25 (24–25)	23 (19–24)
Height (cm)	174 (161–184)	181 (179–184)	168 (161–169)
Weight (kg)	70.3 (59.6–80.8)	75.1 (66.2–80.8)	62.4 (59.6–74.4)
BMI (kg/m^2^)	23.2 (20.2–26.1)	23.4 (20.2–23.9)	23.0 (22.1–26.1)

Data are presented as median with range. BMI, body mass index.

#### Adverse events

The primary endpoint was safety and tolerability of the Filter and Magnet component devices, defined as the incidence of AEs. A total of 28 AEs were reported over the 7-day study period, all of which were transient and classified as mild. 12 AEs (43%) were judged to be causally related to the study protocol, but not to the treatment, the most common being a haematoma around the cannulation sites and dilution of laboratory parameters due to the high volume of fluids given. Five AEs (18%) were ‘possibly’ related to the component devices (a transient increase in iron concentrations) and 8 AEs (29%) were ‘probably’ related to the component devices (a transient increase in chromium or manganese, further detailed below). A list of all AEs is provided in Supplementary Table S6. Overall, the treatment was well-tolerated by all subjects and did not result in any serious AEs.

#### Vital signs

On the experiment day, no relevant changes were observed in body temperature, mean arterial pressure, and heart rate (Supplementary Fig. S2). Furthermore, subjects did not experience any symptoms after initiation of treatment (Supplementary Fig. S3).

#### Laboratory and immunological safety parameters

**Figure 5:**
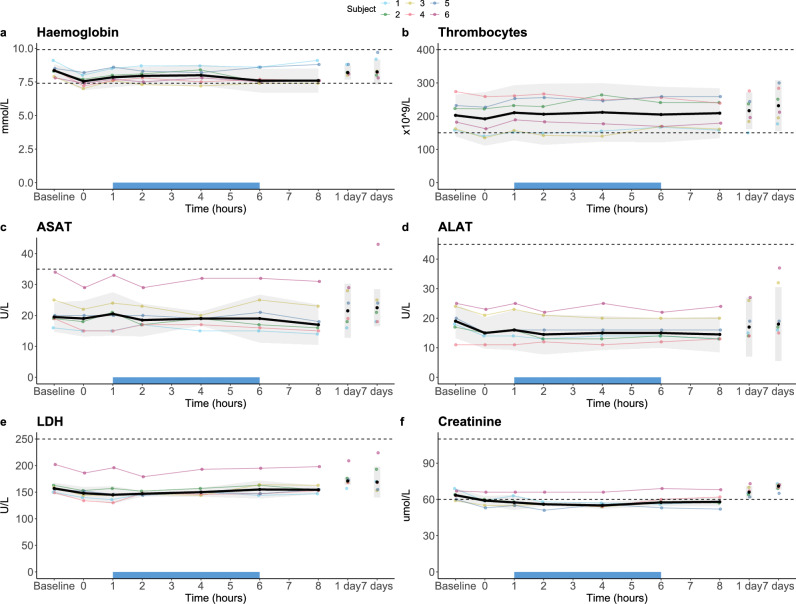
Laboratory safety parameters in the first-in-human study. Data are displayed per individual subject (coloured lines) and as median ± IQR (black line with grey area). The extracorporeal circulation period is indicated by the blue bar on the x-axis. Dashed horizontal lines indicate the reference range. ASAT, aspartate aminotransferase; ALAT, alanine aminotransferase; LDH, lactate dehydrogenase.

**Figure 6:**
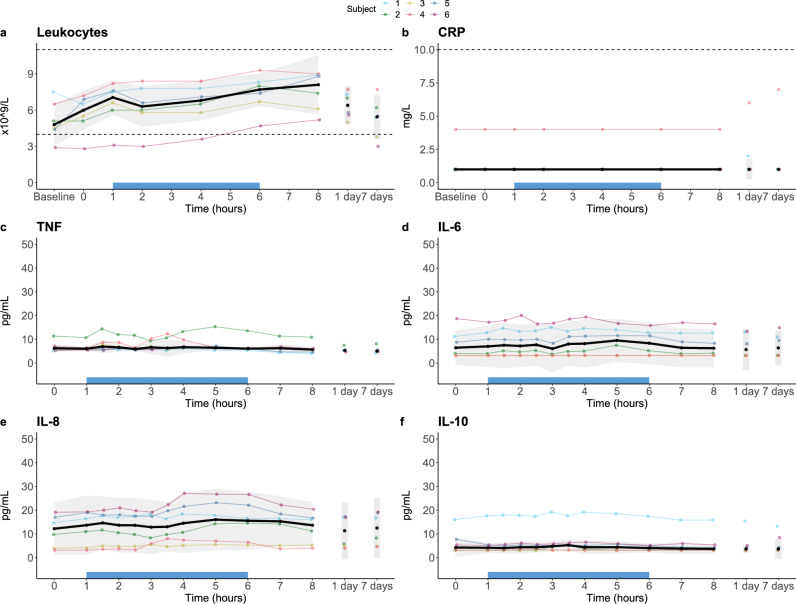
Inflammatory safety parameters in the first-in-human study. Data are displayed per individual subject (coloured lines) and as median ± IQR (black line with grey area). Dashed horizontal lines indicate the reference range. The extracorporeal circulation period is indicated by the blue bar on the x-axis. CRP, C-reactive protein; TNF, tumour necrosis factor; IL, interleukin.

Due to prehydration, a slight decrease in laboratory parameters (e.g. in electrolytes and haemoglobin) was observed at T = 0 h compared to baseline (T = − 1 h). During treatment, from T = 1 to 6 h, haemoglobin and thrombocytes showed no relevant changes (Fig. 5a and b respectively). Liver function enzymes (i.e. ALAT, ASAT, and lactate dehydrogenase (LDH), shown in Fig. 5c–e, respectively), and glomerular filtration rate remained within normal range throughout the study period (Supplementary Table S7). Creatinine (Fig. 5f) slightly decreased over time, again due to the (pre)hydration fluids administered but reverted to normal at the first follow-up visit. Inflammatory parameters are displayed in Fig. 6. Leukocytes showed a slight increase over time, although they remained within the normal range (Fig. 6a), and C-reactive protein (CRP) remained below 10 mg/L throughout the study period (Fig. 6b). Furthermore, plasma concentrations of TNF, IL-6, IL-8 and IL-10 did not relevantly change (Fig. 6c–f). Other laboratory parameters and plasma cytokine concentrations also showed no relevant alterations over time (Supplementary Tables S7 and S8 respectively).

#### Metal analysis

**Figure 7:**
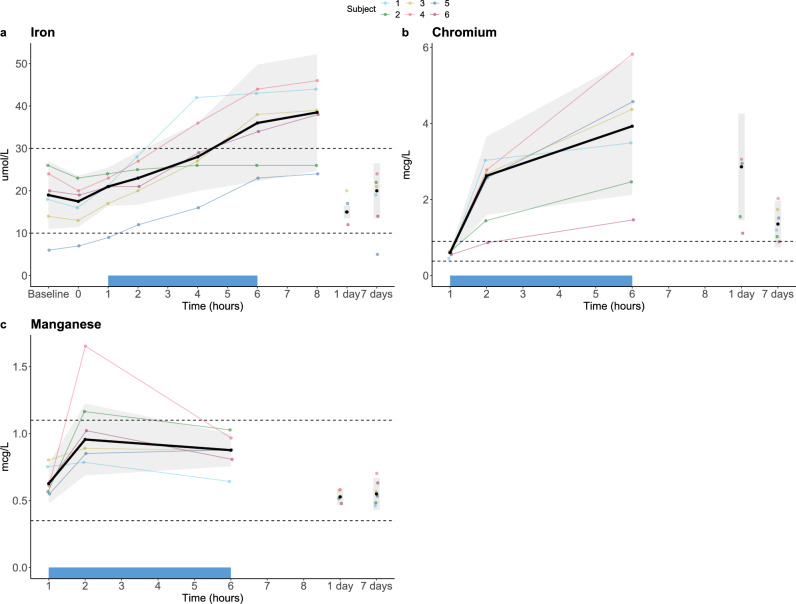
Metal analysis in the first-in-human study. Data are displayed per individual subject (coloured lines) and as median ± IQR (black line with grey area). The extracorporeal circulation period is indicated by the blue bar on the x-axis. Dashed horizontal lines indicate the reference range.

Serum iron increased following the start of treatment, peaking at T = 8 h. Four subjects had a peak iron concentration slightly above the upper limit of normal, however these had returned to normal concentrations at the first follow-up visit (Fig. 7a). Chromium concentrations transiently increased after the initiation of treatment to above the upper limit of normal in all subjects, with the highest plasma concentration of 5.85 µg/L measured at T = 6 h in one subject. Chromium concentrations decreased over time, however they remained slightly elevated above normal concentrations 7 days following treatment for all but one subject, who reverted to normal concentrations (Fig. 7b). For manganese concentrations, a transient increase was observed following initiation of treatment in two subjects (highest measured plasma concentration of 1.65 µg/L at T = 2 h in one subject), but concentrations normalised immediately following cessation of treatment at T = 6 h (Fig. 7c). An independent toxicologist reviewed the chromium and manganese results. In a written report, the toxicologist concluded that there were no adverse health effects and this report was also presented to the data safety and monitoring board (DSMB), which came to the same conclusion. The ethics committee was notified of the DSMB’s conclusion.

## Discussion

In the present study, we evaluated the safety and tolerability of a novel haemofiltration system based on high-gradient magnetic separation—the “IL-6-Sieve” device, comprising Filter, Magnet, Bead Adapter, and Anti-IL-6 Beads component devices—in several animal models and in healthy volunteers. Extracorporeal treatment using the Filter and Magnet was not associated with any serious adverse events or clinically relevant changes in vital signs or laboratory safety parameters in both animals and healthy volunteers. Furthermore, administration of the Anti-IL-6 Beads in sheep, either by infusion into an extracorporeal circuit using the Bead Adapter, or direct by intravenous injection (not the intended clinical use), did not result in haematological or biochemical changes of clinical or veterinary significance. A biodistribution study in mice revealed that intravenous injection of the Anti-IL-6 Beads in mice leads to their accumulation in the liver and spleen, but not in the kidneys or the brain.

In the animal studies, no AE or clotting issues occurred. In the first-in-human study, however, we initially encountered clotting issues when only unfractionated heparin was used, which was resolved when acetylsalicylic acid was added to the anticoagulation regimen. A similar problem occurred in a recent study by our group investigating another haemoadsorption device^21^. In contrast to healthy volunteers, the coagulation response in sepsis patients is altered (e.g. thrombocytopenia or prolonged global coagulation times) and therefore the use of dual anticoagulatory strategies may ultimately not be required for haemofiltration with the IL-6-Sieve in sepsis patients^15,27,28^.

Several adverse events occurred in healthy volunteers, all of which were transient and of mild severity. Almost half of the AEs were related to the study protocol and not to the Filter or Magnet. However, a third of the AEs documented were caused by an increase in chromium or manganese concentrations and were classified as ‘probably related’ to the Filter. The increase in chromium and manganese is likely caused by the stainless-steel layers of mesh inside the Filter, especially as plasma concentrations were already elevated above the normal range before subjects were allowed to eat and drink (both metals are present in nutrition). Furthermore, a fifth of the AEs were due to a small increase in iron. Iron release has been observed before with the use of metal-containing devices, such as metallic prosthetics^29^. However, iron concentrations can also increase due to dietary intake. Therefore, the transient increase observed was classified as ‘possibly related’ to the Filter.

Chromium and manganese are both essential trace minerals in human nutrition. However, high plasma concentrations for a prolonged period of time may result in adverse health effects^30^. An increase in chromium has been observed in patients receiving metal implants, such as metal-on-metal hip arthroplasties or stainless-steel bars for correction of a pectus excavatum^29,31,32^. Importantly, the elevated concentrations observed were not associated with any symptoms. Furthermore, chromium concentrations declined rapidly over time in our study compared to the long period of exposure in the previous studies. For manganese, reported reference values in blood vary, due to differences in geography and whether manganese was measured in whole blood or plasma^33,34^. For instance, a study comparing manganese concentrations in whole blood and plasma reported a reference range of 63–260 nmol/L (i.e. 3.46–14.28 µg/L) and 19–30 nmol/L (i.e. 1.04–1.65 µg/L), respectively, whereas another study reported a broader plasma reference range of 0.3–2.5 µg/L^33,34^. Considering these reference values, the highest manganese concentration measured in our study would still be within normal limits. Thus, although plasma concentrations of chromium and manganese were transiently increased in our study, this is highly unlikely to have caused any adverse health effects.

Although in the intended use of the IL-6-Sieve the extracorporeally-introduced Anti-IL-6 Beads are intended to be captured and retained inside the Filter before the blood is returned to the patient. In both mice and sheep, direct injection of Anti-IL-6 Beads into the circulation was performed to evaluate any effects in the event that some Anti-IL-6 Beads might accidently escape the Filter and enter the circulation in an exaggerated model of administering the same amount of Anti-IL-6 Beads normally used in 4 h within 15 min. The biodistribution study in mice revealed that Anti-IL-6 Beads accumulated in the liver and especially in the spleen, which is unsurprising as both organs play an important role in metabolism and/or immunology^35,36^. Importantly, when the Anti-IL-6 Beads were directly injected into a sheep, no alterations in liver enzymes were observed up until 4 h after direct infusion. That said, although there were no signs of acute toxicity, long term effects cannot be fully ruled out based on this experimental setup.

Of interest, although the Anti-IL-6 Beads used in this study were designed specifically for the purpose of coupling with IL-6 antibodies, the magnetic haemofiltration modality itself has broader applicability, simply by tailoring the surface chemistry of the Beads. For instance, it has potential for the management of diseases that are currently treated by plasmapheresis, such as idiopathic or thrombotic thrombocytopenic purpura, Goodpasture syndrome, myasthenia gravis or Guillain-Barré syndrome^37,38^. The modality could also aid adeno-associated virus (AAV) gene therapy, as AAV transduction and thus the efficacy of gene therapy is hampered by neutralizing antibodies (NAb) which are often present after an infection with wildtype AAV^39^. By removing NAb, the modality has potential to improve AAV gene therapy efficacy.

This study has several limitations that have to be taken into account. First, the biodistribution of Anti-IL-6 Beads was only examined in a small study in mice. Also, these mice were only observed for 24 h, a timepoint at which beads were still present in the liver and spleen. A GLP-compliant study in larger animals, such as sheep, and with longer follow-up, may be required before safety and efficacy studies of these beads in humans can be performed. Second, the first-in-human safety study we performed has a relatively small sample size, therefore rare side-effects might have been left unnoticed.

To conclude, these studies show that the Filter and Magnet used in the IL-6-Sieve device are well-tolerated and without safety concerns in both sheep and healthy volunteers. Further investigation of the safety of the Anti-IL-6 Beads and Bead Adapter in large animals and humans is required before the efficacy of using the IL-6-Sieve device to remove IL-6 from the circulation can be investigated in clinical studies.

### Supplementary Information

Supplementary Information.

## Data Availability

The data generated and analysed during the current study are available from the corresponding author on reasonable request.
