# Supplementary material for: Preclinical and first-in-human safety studies on a novel magnetism-based haemofiltration method
Source: Sci Rep. 2024 Jun 18;14:14077. doi: 10.1038/s41598-024-64379-9 (PMC11189386; doi:10.1038/s41598-024-64379-9)
Supplement: Supplementary file 1 — Supplementary Information. [file 41598_2024_64379_MOESM1_ESM.pdf]

Supplementary Methods

Mechanism of action of the IL-6-Sieve

The Filter works in conjunction with the Magnet. When placed inside the Magnet, a magnetic field is induced in the filtering element. This magnetic field is parallel to the fluid flow through the filtering element. The fluid flowing into the filtering element is slowed owing to the increased cross-sectional area. Magnetic components are attracted to areas of high magnetic flux within the structure of the filtering element and are retained in areas that have lower drag force. Non-magnetic components are not retained due to the pore size and have free passage though the filtering element. The Bead Adapter facilitates the connection between the Filter and the extracorporeal circuit for treatments that require the use of magnetic beads. Through a syringe pump, it enables the introduction of Beads into the system before reaching the Filter.

Regulatory guidelines under which the animal studies were performed

1. Safety of the Filter and Magnet

- This study was conducted under UK Animal (Scientific Procedures) Act 1986 (PPL 7007765).
- All procedures were in compliance with the Good Laboratory Practice Regulations 1999 (S.I. No. 3106) as amended by the 2004 regulations (S.I. No. 994) which are based on the principles of good laboratory practice as adopted by the Organisation for Economic Co-operation and Development (OECD), ENV/MC/CHEM (98) 17. They are in conformity with, and implement the requirements of, EU Directives 2004/09/EC and 2004/10/EC.
- Standard Operating Procedures of the test facility applied.

2. Safety of the Anti-IL-6 Beads and Bead Adapter, and of the IL-6-Sieve

- This study was conducted under UK Animal (Scientific Procedures) Act 1986 under PPL PP9716426.
- Standard Operating Procedures of the test facility applied.

3. Biodistribution of Anti-IL-6 Beads

- Finnish national legislation:
  - This study was conducted under a license for animal experiments approved by the National Animal Experiment Board [8072/2021].
  - All procedures were in compliance with the Act on the Protection of Animals Used for Scientific or Educational Purposes (497/2013) as well as with the Government Decree on the Protection of Animals Used for Scientific or Educational Purposes (564/2013)
- European and international legislation and guidelines:
  - All study procedures complied with Directive 2010/63/EU, Commission recommendation 2007/526/EC, and the Guide for the Care and Use of Laboratory Animals (Guide), Eighth Edition (National Research Council 2011).
- Institutional guidelines:
  - The study protocol was reviewed by the Animal Welfare Team on March 23rd 2022
  - This study was performed according to appropriate methodologies and standard operating procedures (SOPs) at the testing facility or test sites.
  - Internal Audits were regularly performed by CR DS Finland Ethics Committee and Animal Welfare Body (EC/AWB).
- Charles River Discovery Sciences (CR DS) Finland site in Kuopio is accredited by Association for Assessment and Accreditation of Laboratory Animal Care (AAALAC) International.

Study procedures

2. Safety of the Anti-IL-6 Beads and Bead Adapter, and of the IL-6-Sieve

Anaesthesia and cannulation procedures during the first experiment (sheep 4)

A 16G BD AngiocathTM (BD, USA) cannula was inserted under local anaesthesia in the left jugular vein for administration of the Anti-IL6 Beads and frequent blood sampling. Clinical monitoring like animal demeanour, temperature, pulse and respiration rate was performed three times before the start of Anti-IL-6 Bead injection, every 4-6 minutes up until 30 minutes after start administration of Anti-IL-6 Beads, and thereafter every 30 minutes up until euthanasia.

Anaesthesia, cannulation, and monitoring procedures during the second experiment (sheep 5)

Anaesthesia was induced with a combination of ketamine (10 mg/kg) and midazolam (0.5 mg/kg) intravenously. Subsequently, the sheep was orally intubated with an appropriately sized cuffed endotracheal tube under isoflurane anaesthesia. Anaesthesia was maintained with sevoflurane under controlled mechanical ventilation and periprocedural analgesia was established with fentanyl (2 µg/kg/hour). A catheter was placed in the cephalic vein for the administration of Compound Sodium Lactate (5 mL/kg/hour) throughout the procedure, and an arterial cannula was placed in the auricular artery for continuous blood pressure measurements. A 12-14G Fr dual lumen dialysis catheter was aseptically placed in the right jugular vein to allow for extracorporeal circulation on the Aquarius Haemofiltration pump (Nikkiso Aquarius platform, Nikkiso Europe GmbH, Germany). The Filter was primed with 0.9% saline prior to connection to the sheep. Besides blood pressure, the sheep was monitored regularly by pulse oximetry, electrocardiogram and capnograph, and signs of discomfort by the anaesthesiologist.

Sample measurements

Haematology samples were placed on wet ice until analysis on a ADVIA 2120i system (Siemens Healthineers, Germany). Clinical biochemistry and total iron was measured in whole blood samples on a Beckman Coulter AU680 analyser (Beckman Coulter Inc., USA). Blood samples for IL-6 were centrifuged at 1700g for 10 minutes at 4°C, after which plasma was stored at -15°C until analysis. IL-6 plasma samples were analysed in triplicate using an Ovine IL-6 ELISA kit (Invitrogen, Thermo Fisher Scientific, USA), with a minimum detectable IL-6 concentration of 0.15 pg/mL.

3. Biodistribution of Anti-IL-6 Beads

Study procedures

Only mice with plasma bile acid <20 µmol/L were used to exclude animals with portosystemic liver shunt. Prior to blood sampling, animals were habituated to the restraining tubes used in the sampling procedure for 3 days (0.5-1 minute per day). Blood samples were collected from the saphenous vein in lithium-heparin tubes and centrifuged at 2000g for 10 minutes at 4°C within 30 minutes from collection, after which plasma was stored at -80°C until analysis. Plasma bile acid concentrations were analysed on a Thermo Fisher Konelab Xti 20 analyser (Thermo Fisher Scientific, USA).

Magnetic resonance imaging

MRI was performed in a horizontal Bruker BioSpec 11.7 T high-field preclinical MRI scanner with a bore size of 160 mm equipped with a gradient set capable of a maximum gradient strength of 750 mT/m, and interfaced with a Bruker Avance III console (Bruker Biospin GmbH, Germany) and a volume coil with a 25 mm inner diameter (Bruker Biospin GmbH, Germany) as transmitter and receiver (Rapid Biomedical GmbH, Germany). For brain imaging, a volume coil was used for transmission and a two-element surface array coil for receiving (Rapid Biomedical GmbH, Germany). The following scanning parameters were set for abdominal imaging (T2*-weighted 2D FLASH): TR = 400 milliseconds (ms), TE = 3 ms, flip angle = 30 degrees and 8 averages, field of view = 25.6 mm * 25.6 mm, matrix size = 256 * 256, slice thickness = 500 µm, and acquisition time = 15 minutes. The area of interest (liver, kidneys and spleen) and reference region (muscle) was covered using coronal slices and at in-plane resolution of 100 microns. T2-weighted TurboRARE spin echo sequence with the same geometry was acquired with effective TE 20.5 ms, TR 1240 ms, rare factor 8, 8 averages and 5 minutes of scanning time. For brain imaging, the following scanning parameters were set (High resolution T2*-weighted 3D brain images): 3D TR = 50 ms, TE = 6 ms, flip angle = 30 degrees and 1 average with 100x100x100 microns isotropic resolution, and scan time = 14 minutes and 40 seconds. High Resolution 3D multi-gradient-echo (MGE) images with the same isotropic resolution were acquired with TR = 75 ms, 6 different echo times (at 3, 13, and 23 ms) and a scan time of 15 minutes. Susceptibility effect of suspect beads over echoes was examined and anatomical images with high signal to noise ratio and T2*/T1 mixed contrast were created by summing over echo images.

Supplementary Table S1 - Overview of the blood and organ sampling timepoints as well as assessed clinical parameters in the GLP safety study on the IL-6-Sieve's Filter and Magnet component devices in sheep.

Timepoint	Pre-anaesthesia	Pre-heparin	Post-heparin	15 min	30 min	60 min	120 min	Day 1	Day 3	Day 7	Day 14	Day 21	Day 28
				Extracorporeal circulation									
				<u>Pre- & post-filter</u>									
**Haematology**	X	X	X	X	X	X	X	X	X	X	X		X
**Clinical chemistry**	X	X	X	X	X	X	X	X	X	X	X		X
**Osmotic fragility analysis**	X						X[1]	X	X	X	X		X
**Blood smear**	X	X	X	X	X	X	X	X	X	X	X	X	X
**Organ and bone marrow tissue collection**													X
**Body weight measurement**	X							X	X	X	X	X	X
**Food & water consumption**								Daily					
**Clinical observations**	Daily observations of general appearance, physical activity, general appetite, urination, urine appearance, defaecation and faeces appearance												

min = minutes. [1] *= only pre-filter.*

Supplementary Table S2 – Overview of the blood sampling timepoints of the second experiment (sheep 5) of the safety study on the IL-6-Sieve's Anti-IL-6 Beads, Bead Adapter, Filter, and Magnet component devices in sheep.

**Timepoint**		**Pre-anaesthesia**	**Post-heparin bolus**	**0 min**	**30 min**	**60 min**	**120 min**	**180 min**	**240 min**
				**Haemofiltration**					
**Haematology**	Access port	X*	X	X	X	X	X	X	X
	Return port			X	X	X	X	X	X
**Clinical Biochemistry**	Access port	X*	X	X	X	X	X	X	X
**Total Iron**	Access port	X*	X						X
**IL-6 ELISA**	Access port	X*	X	X	X	X	X	X	X

Start of Anti-IL-6 Beads infusion is start of treatment at 0 min.
*min = minutes. * blood is obtained from the cephalic vein.*

Supplementary Table S3 - Body weight throughout the GLP safety study on the IL-6-Sieve's Filter and Magnet component devices in sheep.

Animal number	Study day				
	0	7	14	21	28
**Sheep 1**	81.0 kg	78.0 kg	76.0 kg	77.0 kg	79.0 kg
**Sheep 2**	68.0 kg	67.0 kg	65.0 kg	68.0 kg	70.0 kg
**Sheep 3**	76.0 kg	75.5 kg	77.0 kg	76.5 kg	77.0 kg

kg = kilograms.

Supplementary Table S4 – Laboratory parameters of the GLP safety study on the IL-6-Sieve's Filter and Magnet component devices in sheep.

	Sheep	Pre-anaesthesia	Pre-heparin	Post-heparin	15 min		30 min		60 min		120 min		Day 1	Day 3	Day 7	Day 14	Day 28	Reference range
					Extracorporeal circulation													
					Pre-filter	Post-filter	Pre-filter	Post-filter	Pre-filter	Post-filter	Pre-filter	Post-filter						
**Haemoglobin (mmol/L)**	1	6.7	5.7	5.0#	4.5#	4.4#	4.5#	4.5#	4.4#	4.3#	4.5#	4.5#	6.3	6.2	6.1	5.9	5.6	5.5-9.6
	2	7.9*	5.0#	4.7#	4.0#	4.1#	4.0#	4.3#	3.8#	4.1#	4.0#	3.9#	6.9	7.4	6.3	7.0	6.8	
	3	8.1	6.2	5.7	5.4#	5.5	5.4#	5.5	5.3#	5.3#	5.2#	5.4#	8.1	7.3	7.6	7.6	7.1	
**Haematocrit (L/L)**	1	0.327	0.295	0.253#	0.214#	0.215#	0.218#	0.212#	0.216#	0.220#	0.219#	0.217#	0.310	0.310	0.301	0.294	0.274	0.258-0.440
	2	0.393*	0.234#	0.217#	0.197#	0.199#	0.194#	0.197#	0.196#	0.194#	0.193#	0.194#	0.343	0.375	0.324	0.353	0.340	
	3	0.399	0.306	0.278	0.264	0.259	0.257#	0.260	0.255#	0.256#	0.249#	0.246#	0.408	0.372	0.385	0.389	0.357	
**Thrombocytes (x10^9/L)**	1	386	215#	241#	232#	160#	187#	104#	204#	202#	187#	186#	287	450	540	388	360	247.3-764.8
	2	83*,#	239#	220#	200#	191#	218#	199#	222#	193#	188#	205#	328	319	413	261	260	
	3	556	391	388	288	120#	235#	119#	262	312	269	250	542	453	593	504	425	
**Leukocytes (x10^9/L)**	1	13.05	10.69	11.43	11.65	10.26	12.17	10.07	11.62	12.01	12.63	12.20	14.21#	11.28	11.07	12.54	12.62	4.0-13.8
	2	8.81*	8.03	9.42	10.36	10.04	10.64	10.62	10.27	9.89	10.75	10.74	11.18	9.68	9.42	9.94	9.76	
	3	11.38	9.22	9.18	7.97	6.96	8.59	7.48	8.02	8.20	9.79	9.53	11.68	9.19	8.47	9.27	9.81	
**Red blood cell count (x10^9/L)**	1	10.38	9.06	7.83#	6.83#	6.83#	6.93#	6.93#	6.87#	6.98#	7.12#	6.98#	9.82	9.76	9.49	9.27	8.72#	8.8-16.0
	2	12.7*	7.59#	7.09#	6.53#	6.56#	6.45#	6.48#	6.56#	6.46#	6.46#	6.51#	11.24	11.97	10.58	11.34	11.14	
	3	11.93	9.18	8.45#	7.92#	7.93#	7.78#	7.98#	7.80#	7.78#	7.63#	7.51#	12.19	11.14	11.55	11.65	10.88	
**MCV (fL)**	1	31.5	32.6	32.3	31.3	31.4	31.5	30.7	31.4	31.5	30.8	31.1	31.6	31.8	31.7	31.7	31.4	21.6-34.9
	2	30.9*	30.8	30.7	30.2	30.4	30.1	30.3	29.8	30.0	29.8	29.8	30.5	31.3	30.7	31.1	30.5	
	3	33.5	33.3	32.9	33.3	32.7	33.0	32.6	32.7	32.9	32.7	32.7	33.5	33.4	33.3	33.4	32.9	
**MCH (pg)**	1	10.4	10.1	10.4	10.5	10.4	10.3	10.3	10.3	10.1	10.2	10.4	10.4	10.3	10.3	10.2	10.3	8.3-12.3
	2	10.1*	10.7	10.6	10.0	10.1	10.1	10.6	9.5	10.3	9.9	9.7	9.9	10.0	9.6	9.9	9.9	
	3	10.9	10.9	10.8	11.0	11.1	11.2	11.1	10.9	11.1	10.9	11.6	10.8	10.6	10.7	10.5	10.6	
**MCHC (g/dL)**	1	33.0	31.1#	32.1#	33.6	33.0	32.8	33.7	32.8	32.0#	33.2	33.4	33.0	32.4#	32.5#	32.2#	32.9	

	2	32.6*,#	34.9	34.6	33.2	33.3	33.5	34.9	31.8#	34.1	33.3	32.7	32.5#	32.0#	31.4#	31.8#	32.3#	32.7-37.3
	3	32.6#	32.8	32.9	33.1	33.9	33.9	34.2	33.3	33.6	33.4	35.6	32.2#	31.7#	32.0#	31.4#	32.3#	
**MPV (fL)**	1	8.9#	7.5	7.2	7.0	7.8	6.7	8.1	6.9	7.5	7.5	7.1	6.6	9.0#	7.4	6.1	7.5	4.4-8.1
	2	12.6*,#	6.3	5.8	6.0	5.9	5.9	5.8	5.9	6.0	6.3	5.7	6.3	6.5	8.5	5.7	6.4	
	3	6.8	4.8	4.8	5.1	7.3	5.4	7.7	5.5	5.9	5.8	6.3	7.5	7.9	6.3	5.5	6.2	
**Neutrophils ($x10^9/L$)**	1	4.72	3.37	3.77	5.12	3.81	5.45	3.79	5.17	5.34	6.24#	5.54	5.66	3.25	2.49	2.66	2.76	1.4-6.0
	2	1.97*	1.51	2.18	3.20	2.95	3.41	3.33	3.37	3.11	3.93	3.80	3.11	2.38	1.85	1.89	2.07	
	3	2.80	2.13	1.94	1.74	1.08#	2.34	1.48	2.11	2.13	3.66	3.30	4.07	1.95	1.97	2.03	2.92	
**Neutrophils (%)**	1	36.2	31.5	33.0	43.9	37.2	44.8	37.6	44.4	44.4	49.4#	45.4	39.8	28.8	22.5	21.2	21.9	9.6-48.7
	2	22.3*	18.8	23.1	30.9	29.4	32.0	31.4	32.8	31.4	36.6	35.4	27.8	24.6	19.6	19.0	21.2	
	3	24.6	23.1	21.1	21.8	15.5	27.3	19.8	26.3	26.0	37.4	34.6	34.9	21.3	23.3	21.9	29.8	
**Lymphocytes ($x10^9/L$)**	1	6.58	6.24	6.45	5.25	5.44	5.50	5.46	5.40	5.52	5.16	5.52	6.77	6.47	7.29	7.49	7.75	2.0-9.5
	2	4.87*	4.84	5.52	5.41	5.31	5.42	5.47	5.30	5.28	5.30	5.46	5.74	5.41	6.26	6.53	6.36	
	3	6.47	5.55	5.66	5.13	5.01	5.17	5.01	4.82	5.06	4.96	4.80	6.10	5.74	5.33	5.58	5.17	
**Lymphocytes (%)**	1	50.4	58.4	56.5	45.1	53.0	45.2	54.2	46.4	45.9	40.8	45.3	47.7	57.4	65.9	59.7	61.4	36.9-72.1
	2	55.2*	60.3	58.6	52.3	52.9	51.0	51.5	51.6	53.4	49.3	50.8	51.4	55.9	66.5	65.7	65.2	
	3	56.8	60.2	61.7	64.3	71.9	60.2	67.0	60.1	61.8	50.7	50.4	52.2	62.4	62.9	60.2	52.7	
**Monocytes ($x10^9/L$)**	1	0.34	0.10	0.14	0.37	0.22	0.26	0.20	0.15	0.20	0.46	0.09	0.13	0.11	0.21	0.30	0.14	0-0.9
	2	0.12*	0.10	0.08	0.16	0.10	0.08	0.14	0.11	0.08	0.19	0.07	0.16	0.27	0.14	0.17	0.25	
	3	0.34	0.28	0.25	0.09	0.10	0.12	0.18	0.14	0.13	0.16	0.22	0.19	0.20	0.14	0.18	0.36	
**Monocytes (%)**	1	2.6	0.9	1.2	3.2	2.1	2.1	2.0	1.3	1.7	3.7	0.7	0.9	1.0	1.9	2.4	1.1	0.3-7.3
	2	1.4*	1.2	0.8	1.5	1.0	0.8	1.3	1.1	0.8	1.8	0.7	1.5	2.8	1.4	1.8	2.5	
	3	3.0	3.1	2.7	1.1	1.4	1.4	2.4	1.8	1.6	1.7	2.3	1.6	2.2	1.7	1.9	3.7	
**Eosinophils ($x10^9/L$)**	1	1.15	0.79	0.87	0.78	0.67	0.81	0.52	0.72	0.81	0.57	0.83	1.44#	1.2	0.93	1.98#	1.79#	0-1.3
	2	1.80*,#	1.46#	1.53#	1.45#	1.58#	1.61#	1.57#	1.39#	1.34#	1.22	1.33#	2.00#	1.52#	1.10	1.26	0.95	
	3	1.57#	1.08	1.14	0.88	0.67	0.80	0.73	0.81	0.76	0.86	1.07	1.19	1.17	0.91	1.32#	1.19	
**Eosinophils (%)**	1	8.8	7.4	7.6	6.7	6.6	6.7	5.2	6.2	6.7	4.5	6.8	10.2#	10.6#	8.4#	15.8#	14.2#	0-8.2
	2	20.5*,#	18.2#	16.3#	14.0#	15.8#	15.1#	14.8#	13.5#	13.6#	11.4#	12.4#	17.9#	15.7#	11.7#	12.7#	9.7#	
	3	13.8#	11.7#	12.4#	11.1#	9.6#	9.3#	9.8#	10.1#	9.3#	8.8#	11.2#	10.1#	12.7#	10.8#	14.3#	12.2#	
**Basophils ($x10^9/L$)**	1	0.24#	0.10	0.10	0.10	0.11	0.13	0.10	0.15	0.14	0.15	0.19	0.14	0.16	0.09	0.09	0.10	0-0.2
	2	0.04*	0.07	0.05	0.07	0.05	0.06	0.06	0.06	0.05	0.06	0.05	0.09	0.06	0.06	0.06	0.06	

	3	0.13	0.11	0.10	0.07	0.07	0.08	0.07	0.07	0.08	0.08	0.10	0.07	0.09	0.07	0.10	0.10	
**Basophils (%)**	1	1.8#	0.9	0.9	0.8	1.1	1.1	1.0	1.3	1.1	1.2	1.5	1.0	1.4	0.9	0.8	0.8	0-1.7
	2	0.4*	0.8	0.5	0.6	0.5	0.6	0.5	0.6	0.5	0.6	0.5	0.8	0.6	0.6	0.6	0.6	
	3	1.1	1.2	1.1	0.9	1.1	0.9	1.0	0.9	1.0	0.8	1.1	0.6	1.0	0.9	1.1	1.1	
**Large unstained cells (x10^9/L)**	1	0.02	0.08	0.08	0.03	0.01	0.01	0.01	0.04	0.01	0.04	0.04	0.06	0.08	0.05	0.02	0.09	0-0.2
	2	0.02*	0.05	0.06	0.06	0.04	0.06	0.05	0.04	0.03	0.04	0.03	0.08	0.04	0.01	0.03	0.08	
	3	0.07	0.06	0.09	0.07	0.04	0.08	0.01	0.06	0.03	0.06	0.04	0.06	0.04	0.04	0.06	0.06	
**Large unstained cells (%)**	1	0.2	0.8	0.7	0.2	0.1	0.1	0.1	0.3	0.1	0.4	0.4	0.5	0.7	0.5	0.2	0.7	0-3.4
	2	0.2*	0.6	0.6	0.6	0.4	0.5	0.4	0.4	0.3	0.4	0.3	0.7	0.4	0.1	0.3	0.8	
	3	0.6	0.7	1.0	0.9	0.5	0.9	0.2	0.8	0.3	0.7	0.4	0.5	0.5	0.4	0.6	0.7	
**Urea (mmol/L)**	1	5.0	5.4	5.4	5.5	5.1	5.5	5.4	5.3	5.5	5.4	5.7	4.6	6.7	4.5	4.9	4.4	1.7-8.5
	2	5.7	5.6	5.6	5.7	5.4	5.6	5.8	5.7	5.6	5.7	5.8	6.1	6.6	4.9	4.9	4.6	
	3	5.1	5.1	5.2	5.1	5.1	5.1	4.8	5.2	5.1	5.2	5.1	6.9	5.8	4.1	3.6	4.3	
**Creatinine (µmol/L)**	1	119	113	113	111	110	113	110	111	112	119	117	118	108	124	134	117	59-155
	2	125	116	117	117	113	116	113	118	117	119	119	124	110	123	126	122	
	3	111	107	106	104	105	105	104	103	102	103	106	113	106	125	127	106	
**Creatine kinase (U/L)**	1	83	173	195	274	274	302	300	349	350	459#	451#	637#	927#	60	79	60	<414
	2	106	153	162	213	210	227	233	283	278	377	377	871#	589#	75	62	77	
	3	116	196	455#	605#	599#	696#	686#	836#	818#	836#	1008#	784#	267	98	97	88	
**ASAT (U/L)**	1	87	80	79	69	74	72	73	76	74	75	74	192	213	103	69	59	25-225
	2	97	84	83	80	79	79	79	79	71	81	80	145	171	97	74	83	
	3	92	87	86	83	84	84	84	87	86	87	90	373#	309#	131	87	77	
**ALAT (U/L)**	1	12	14	13	11	12	13	12	16	12	10	13	26	35	22	14	13	4-36
	2	16	14	14	13	13	13	14	13	13	13	13	23	28	20	15	20	
	3	19	19	18	18	17	18	17	17	17	17	18	52#	51#	32	22	20	
**Alkaline phosphatase (U/L)**	1	40	29	27	29	28	29	28	26	27	23	25	44	44	40	57	49	<206
	2	64	65	55	46	47	47	45	49	48	48	50	58	65	47	47	79	
	3	36	26	28	31	33	29	29	33	34	33	31	44	26	25	38	49	
**Inorganic phosphorus (mmol/L)**	1	2.0	2.2	2.2	2.2	2.2	2.1	2.1	2.1	2.1	2.1	2.1	1.7	1.7	1.5	1.4	1.4	0.6-3
	2	1.7	1.9	1.9	1.8	1.7	1.8	1.7	1.9	1.8	2.0	2.0	1.3	1.9	1.4	1.3	1.5	
	3	2.6	2.5	2.4	2.4	2.4	2.4	2.5	2.4	2.5	2.4	2.5	2.9	2.2	2.1	1.9	2.0	

**Total protein (g/L)**	1	81.7	74.4	73.2	68.9	67.2	67.5	67.6	67.3	66.9	67.6	66.9	74.1	76.5	76.1	75.6	72.4	44-92
	2	75.8	66.9	64.9	62.2	61.8	60.6	60.9	60.5	60.3	59.9	60.4	73.4	74.2	69.2	70.1	70.9	
	3	74.3	71.0	66.7	63.6	63.8	63.8	63.0	62.3	61.8	62.3	61.6	74.7	71.3	71.7	71.4	69.8	
**Albumin (g/L)**	1	27.7	25.9	25.1	23.8	23.7	23.0	24.4	23.6	23.0	23.5	23.8	25.7	25.9	25.9	26.9	25.9	20.8-41.2
	2	32.0	27.8	26.8	26.5	26.7	26.3	25.8	25.8	25.7	26.1	25.8	30.7	31.1	28.9	29.4	29.3	
	3	35.6	34.0	32.3	30.9	30.5	30.4	30.7	31.1	30.1	31.1	29.8	35.9	33.4	33.8	33.7	30.8	
**GGT (U/L)**	1	45	38	37	35	36	35	35	36	36	36	36	84	81	69	60	54	6-94
	2	54	42	40	40	40	40	39	38	39	39	39	51	55	55	53	54	
	3	36	29	28	27	28	28	28	28	28	28	28	35	36	33	32	32	
**Calcium (mmol/L)**	1	2.29	2.16	2.17	2.18	2.14	2.23	2.17	2.20	2.18	2.17	2.20	2.32	2.39	2.35	2.41	2.43	1.4-3
	2	2.31	2.26	2.35	2.36	2.41	2.38	2.39	2.36	2.40	2.39	2.37	2.40	2.38	2.35	2.31	2.41	
	3	2.49	2.46	2.56	2.55	2.48	2.51	2.52	2.56	2.52	2.56	2.53	2.49	2.36	2.27	2.46	2.31	

** = clotted blood sample. # = Values are outside of the normal range. MCV = mean corpuscular volume; MCH = mean cell haemoglobin; MCHC = mean cell haemoglobin concentration; MPV = mean platelet volume; ASAT = aspartate aminotransferase; ALAT = alanine aminotransferase; GGT = gamma-glutamyltransferase.*

Supplementary Table S5 – Laboratory parameters of the animal studies on the safety study on the IL-6-Sieve's Anti-IL-6 Beads, Bead Adapter, Filter, and Magnet component devices in sheep.

	Sheep	Pre-anaesthesia	Post-heparin	0 min		30 min		60 min		120 min		180 min		240 min		Reference range
				Haemofiltration												
				Pre-filter[a]	Post-filter	Pre-filter[a]	Post-filter	Pre-filter[a]	Post-filter	Pre-filter[a]	Post-filter	Pre-filter[a]	Post-filter	Pre-filter[a]	Post-filter	
**Haemoglobin (mmol/L)**	4			5.1[#]		5.4[#]				5.9				5.6		5.6-9.3
	5	7.3	5.6	5.3[#]	5.4[#]	5.8	5.3[#]	5.3[#]	5.2[#]	5.2[#]	5.1[#]	4.9[#]	4.9[#]	5.1[#]	5.0[#]	
**Haematocrit (%)**	4			22.6[#]		24.1[#]				26.9[#]				25.0[#]		27.0-45.0
	5	33.0	24.6[#]	24.1[#]	24.1[#]	24.3[#]	23.3[#]	23.6[#]	23.1[#]	23.0[#]	22.5[#]	22.3[#]	22.3[#]	22.8[#]	22.6[#]	
**Thrombocytes (x10^9/L)**	4			299		318				312				311		nr
	5	295	271	236	157	218	201	208	203	234	232	206	195	214	213	
**Leukocytes (x10^9/L)**	4			6.2		6.6				6.2				5.5		4.0-12.0
	5	6.0	6.3	6.7	6.7	4.9	3.7[#]	5.0	3.8[#]	4.3	3.6[#]	3.8[#]	3.5[#]	4.1	3.3[#]	
**Red blood cell count (x10^9/L)**	4			7.6[#]		8.0[#]				8.8[#]				8.2[#]		9.0-15.0
	5	10.7	8.2[#]	8.1[#]	8.0[#]	8.2[#]	7.8[#]	8.0[#]	7.8[#]	7.8[#]	7.6[#]	7.5[#]	7.4[#]	7.6[#]	7.6[#]	
**MCV (fL)**	4			29.9		30.3				30.4				30.7		28.0-40.0
	5	30.9	30.1	29.7	30.0	29.7	29.9	29.7	29.8	29.6	29.5	29.7	30.0	29.9	29.9	
**MCHC (g/dL)**	4			36.4[#]		36.0[#]				35.5[#]				36.1[#]		31.0-34.0
	5	35.3[#]	36.5[#]	35.8[#]	36.1[#]	38.0[#]	36.4[#]	36.1[#]	36.4[#]	36.1[#]	36.2[#]	35.4[#]	35.5[#]	35.8[#]	35.9[#]	
**Neutrophils (x10^9/L)**	4			1.4		2.2				2.8				2.5		0.7-6.0
	5	1.4	2.8	3.3	3.1	2.0	1.3	2.1	1.4	1.6	1.3	1.6	1.3	1.5	1.2	
**Neutrophils (%)**	4			22.5		33.1				44.8				46.1		nr
	5	23.3	44.1	48.8	47.0	40.9	34.1	41.8	37.2	38.7	34.3	41.1	37.8	36.9	37.0	
**Lymphocytes (x10^9/L)**	4			3.7		3.6				2.8				2.0		2.0-9.0
	5	2.8	2.1	2.0	2.1	1.9[#]	1.6[#]	1.9[#]	1.6[#]	1.5[#]	1.7[#]	1.4[#]	1.0[#]	1.6[#]	1.0[#]	
**Lymphocytes (%)**	4			60.2		53.8				45.1				36.9		nr
	5	46.0	33.7	30.1	31.1	39.0	43.0	38.1	41.0	36.0	46.9	35.9	27.7	39.8	31.2	
**Monocytes (x10^9/L)**	4			0.2		0.2				0.2				0.6		0-0.8
	5	0.1	0.2	0.3	0.2	0.2	0.4	0.2	0.4	0.3	0.2	0.2	0.5	0.3	0.4	

**Monocytes (%)**	4			3.5		2.4				2.9				10.8		nr
	5	1.4	2.9	3.7	3.5	3.2	9.9	3.2	10.0	7.5	4.8	4.8	15.6	7.2	11.6	
**Eosinophils ($x10^9$/L)**	4			0.8		0.7				0.4				0.3		0-1.0
	5	1.7[#]	1.2[#]	1.2[#]	1.2[#]	0.8	0.5	0.8	0.4	0.7	0.5	0.7	0.6	0.6	0.6	
**Eosinophils (%)**	4			13.3		10.3				6.9				5.3		nr
	5	27.9	19.2	17.2	18.3	16.4	12.2	16.6	10.9	17.1	13.3	17.6	18.0	15.1	18.8	
**Basophils ($x10^9$/L)**	4			0.02		0.02				0.02				0.02		nr
	5	0.03	0.01	0.01	0.01	0.01	0.02	0.01	0.01	0.01	0.03	0.02	0.01	0.01	0.01	
**Basophils (%)**	4			0.4		0.3				0.3				0.4		nr
	5	0.5	0.1	0.2	0.1	0.2	0.5	0.2	0.3	0.3	0.7	0.5	0.3	0.3	0.4	
**Urea (mmol/L)**	4			2[#]		2[#]				2[#]				2[#]		3-10
	5	3	3	3		3		3		3		3		3		
**Creatinine (µmol/L)**	4			71		70				67[#]				61[#]		70-105
	5	62[#]	68[#]	67[#]		68[#]		66[#]		65[#]		62[#]		60[#]		
**Creatine kinase (U/L)**	4			344		426				402				346		nr
	5	113	103	113		127		127		154		147		158		
**ASAT (U/L)**	4			123		128				124				126		60-280
	5	135	115	112		110		108		107		103		104		
**ALAT (U/L)**	4			25		26				25				25		22-38
	5	21[#]	18[#]	17[#]		17[#]		17[#]		17[#]		16[#]		16[#]		
**Inorganic phosphorus (mmol/L)**	4			1.47[#]		1.28[#]				1.47[#]				1.18[#]		1.62-2.36
	5	2.03	2.14	2.03		1.88		1.82		1.70		1.51[#]		1.39[#]		
**Total protein (g/L)**	4			72		73				71				70		60-79
	5	72	63	62		61		60		59[#]		57[#]		58[#]		
**Albumin (g/L)**	4			30		30				29				29		24-30
	5	32[#]	28	27		27		27		26		26		26		
**GGT (U/L)**	4			17[#]		8[#]				17[#]				12[#]		20-52
	5	57[#]	49	48		46		47		45		43		45		
**Calcium (mmol/L)**	4			2.60[#]		2.59[#]				2.44[#]				2.52[#]		2.88-3.20
	5	2.50[#]	2.30[#]	2.28[#]		2.23[#]		2.23[#]		2.16[#]		2.16[#]		2.14[#]		
	4			150		149				151				149		145-152

**Sodium (mmol/L)**	5	148	148	147		147		147		147		146		146		
**Potassium (mmol/L)**	4			4.0		4.1				4.0				3.9		3.9-5.4
	5	4.6	4.3	4.3		4.3		4.3		4.0		3.8#		3.8#		
**Chloride (mmol/L)**	4			107#		109#				111#				111#		95-103
	5	108#	107#	108#		107#		107#		107#		107#		107#		
**Globulin (g/L)**	4			41.7		42.5				41.8				40.3		nr
	5	40	35	34		34		33		33		32		32		
**BHB (mmol/L)**	4			0.36#		0.36#				0.54				0.57		0.47-0.63
	5	0.45#	0.21#	0.18#		0.13#		0.12#		0.12#		0.12#		0.10#		
**LDH (U/L)**	4			1253#		1371#				1278#				1251#		240-440
	5	1641#	1377#	1365#		1345#		1334#		1292#		1267#		1300#		
**Total iron (µmol/L)**	4			20#										18#		30-40
	5	27#	21#											23#		
**GLDH**	4															nr
	5	73.5	63.4	60.6		57.7		49.0		47.5		46.0		47.3		
**Interleukin-6 (pg/mL)**	4			0.598		0.389				0.342				0.328		nr
	5	1.314	2.046	1.126		0.967		1.277		1.312		1.475		1.227		

# = Values marked are outside of the normal range. [a] = For sheep 4, no Filter was used, so Pre-filter values represent normal blood draws. Sheep 4 = direct injection of Anti-IL-6 Beads into the circulation without extracorporeal circulation or administration of anaesthesia and heparin; Sheep 5 = haemofiltration and infusion of Anti-IL-6 Beads into the extracorporeal circulation system via the Bead Adapter.

nr = no reference range available; MCV = mean corpuscular volume; MCH = mean cell haemoglobin; MCHC = mean cell haemoglobin concentration; MPV = mean platelet volume; ASAT = aspartate aminotransferase; ALAT = alanine aminotransferase; GGT = gamma-glutamyltransferase; BHB = ß-hydroxybutyrate; LDH = lactate dehydrogenase; GLDH = glutamate dehydrogenase.

Supplementary Table S6 – Number of adverse events in the first-in-human study.

	Frequency of AE
**General disorders**	0
**Nervous system**	0
**Cardiovascular**	0
**Gastrointestinal**	0
**Infectious**	0
**Musculoskeletal**	0
**Dermatological**	
- Haematoma around the cannulation site	6
**Laboratory abnormalities**	
- Dilution of laboratory parameters after prehydration and during subsequent hydration (e.g. haemoglobin, haematocrit, erythrocytes, thrombocytes, sodium, potassium, calcium, phosphate, magnesium, urea, creatinine, albumin)	6
- Increased levels of prothrombin time at baseline (before administration of anticoagulants)	1
- Low serum iron and high red cell distribution width at baseline probably due to a vegetarian diet	1
- Increased levels of creatinine kinase (CK) at baseline and at T = 7 days, which is probably due to extensive exercise the day before the experiment day and the last follow-up visit	1
- Increased levels of serum iron	5
- Increased levels of plasma chromium	6
- Increased levels of plasma manganese	2

Supplementary Table S7 – Laboratory safety parameters of the healthy volunteers in the first-in-human study.

	Baseline	0 hours	1 hour	2 hours	4 hours	5 hours	6 hours	8 hours	Day 1	Day 7	Reference range
	Heparin infusion										
			Extracorporeal circulation								
**Haemoglobin (mmol/L)**	8.4 [8-8.5]	7.6 [7.2-7.9]	7.8 [7.6-8.4]	7.9 [7.6-8.2]	8 [7.6-8.4]		7.6 [7.5-8.4]	7.6 [7.6-8.5]	8.2 [8.1-8.7]	8.2 [8.1-9]	Female: 7.4 - 9.9<br>Male: 8.4 - 10.8
**Haematocrit (L/L)**	0.38# [0.38-0.41]	0.36# [0.35-0.38]	0.37 # [0.37-0.39]	0.37# [0.36-0.4]	0.36# [0.36-0.39]		0.36# [0.35-0.39]	0.36# [0.36-0.4]	0.39# [0.39-0.41]	0.41 [0.4-0.44]	0.41 - 0.53
**Leukocytes (x10^9/L)**	4.8 [4.4-6.2]	6 [5.2-6.8]	7 [6.2-7.6]	6.3 [5.8-7.5]	6.8 [6-7.6]		7.7 [6.9-8.2]	8.1 [6.4-8.9]	6.4 [5.6-7.2]	5.4 [4.2-6]	4.0 - 11.0
**Erythrocytes (x10^9/L)**	4.19# [4.03-4.56]	3.91# [3.8-4.16]	4.06# [4.03-4.38]	4.01# [3.95-4.44]	3.96# [3.91-4.44]		3.96# [3.93-4.37]	4.02# [3.96-4.48]	4.27# [4.19-4.52]	4.42# [4.17-4.8]	4.50 - 5.90
**Thrombocytes (x10^9/L)**	202 [167-230]	192 [146-226]	210 [165-248]	206 [158-249]	212 [160-248]		205 [169-252]	209 [166-240]	216 [187-242]	232 [199-276]	150 - 400
**MCV (fL)**	90 [88-92]	90 [90-92]	90 [90-92]	90 [90-91]	90 [89-91]		89 [88-92]	90 [88-92]	92 [90-92]	92 [90-93]	80 - 100
**MCH (fmol)**	1.96 [1.91-1.98]	1.87 [1.85-1.89]	1.9 [1.87-1.91]	1.9 [1.86-1.92]	1.89 [1.86-1.96]		1.9 [1.88-1.95]	1.9 [1.88-1.94]	1.9 [1.88-1.96]	1.87 [1.86-1.89]	nr
**MCHC (mmol/L)**	21.5 [20.7-22]	20.7 [20.5-21]	20.8 [20.6-21.1]	21 [20.6-21.3]	21 [20.6-21.6]		21.1 [20.8-21.4]	20.9 [20.8-21.4]	20.9 [20.7-21.2]	20.6 [20.2-20.8]	20.0 - 21.5
**RCDW (%)**	12.6 [12.1-13]	12.6 [12.2-12.9]	12.6 [12.1-13]	12.5 [12.1-12.8]	12.4 [12-12.9]		12.4 [12-12.9]	12.6 [12.2-13]	12.7 [12.2-13.1]	12.5 [12.1-13.1]	12.0 - 13.6

**Neutrophilic granulocytes ($x10^9$/L)**	2.8 [2.49-3.5]	3.86 [3-4.77]	4.35 [3.19-5.22]	3.89 [3.05-4.61]	4.12 [3.14-4.5]		4.66 [3.74-4.92]	4.36 [3.46-5.6]	4.31 [3.64-4.67]	3.19 [2.04-3.86]	2.00 - 7.50
**Lymphocytes ($x10^9$/L)**	1.36 [1.24-1.48]	1.4 [1.26-1.66]	1.92 [1.52-2.28]	1.77 [1.49-2.32]	1.97 [1.67-2.23]		2.15 [1.7-2.53]	2.12 [1.75-2.41]	1.41 [1.17-1.77]	1.57 [1.56-1.59]	1.00 - 3.50
**Monocytes ($x10^9$/L)**	0.48 [0.39-0.51]	0.45 [0.34-0.51]	0.52 [0.42-0.58]	0.48 [0.39-0.51]	0.54 [0.45-0.57]		0.52 [0.45-0.59]	0.59 [0.56-0.65]	0.5 [0.41-0.58]	0.37 [0.35-0.38]	0.30 - 1.00
**Eosinophilic granulocytes ($x10^9$/L)**	0.06 [0.05-0.12]	0.07 [0.05-0.09]	0.06 [0.06-0.09]	0.05 [0.04-0.06]	0.05 [0.05-0.06]		0.07 [0.06-0.08]	0.09 [0.07-0.1]	0.08 [0.06-0.13]	0.11 [0.07-0.13]	<0.50
**Basophilic granulocytes ($x10^9$/L)**	0.03 [0.02-0.04]	0.03 [0.03-0.05]	0.04 [0.03-0.04]	0.03 [0.03-0.04]	0.04 [0.03-0.05]		0.04 [0.03-0.04]	0.04 [0.04-0.05]	0.04 [0.03-0.04]	0.03 [0.03-0.05]	<0.10
**Prothrombin time (sec)**	12.6 [12.4-12.9]	14.9$^{\#}$ [14.1-15.3]	15.2$^{\#}$ [14.7-15.5]	15.1$^{\#}$ [14.6-15.8]	15.8$^{\#}$ [15.2-16]		15.1$^{\#}$ [14.9-15.3]	13.9$^{\#}$ [13.7-14]	12.5 [12.3-12.6]	12.2 [11.4-12.5]	10.2 - 13.3
**APTT (sec)**	28 [27-28]	400$^{\#}$ [400-400]	400$^{\#}$ [400-400]	400$^{\#}$ [400-400]	400$^{\#}$ [400-400]		400$^{\#}$ [400-400]	106$^{\#}$ [88-137]	28 [27-29]	28 [28-29]	25 - 38
**APTT ratio**		14.6 [14.3-14.8]	14.3 [14.3-14.8]	14.6 [14.3-14.8]	14.6 [14.3-14.8]		14.6 [14.3-14.8]	3.8 [3.2-4.7]	1 [1-1.1]	1 [1-1]	nr
**Fibrinogen (g/L)**	1.9 [1.6-2.2]	1.7$^{\#}$ [1.5-2]	1.6$^{\#}$ [1.6-2.2]	1.8 [1.6-2]	1.8 [1.6-2.1]		1.7$^{\#}$ [1.6-2]	1.8 [1.7-2]	2.3 [2.1-2.5]	2.2 [1.9-2.4]	1.8 - 4.2
**International Normalized Ratio (INR)**	1 [1-1.1]	1.2 [1.2-1.3]	1.3$^{\#}$ [1.2-1.3]	1.2 [1.2-1.3]	1.3$^{\#}$ [1.2-1.3]		1.2 [1.2-1.3]	1.2 [1.1-1.2]	1 [1-1]	1 [0.9-1]	0.8 - 1.2
**Sodium (mmol/L)**	140 [138-140]	135 [134-137]	139 [138-140]	140 [138-142]	139 [138-141]		140 [138-141]	138 [137-140]	138 [137-139]	140 [139-140]	135 - 145

**Potassium (mmol/L)**	3.8 [3.7-3.8]	3.6 [3.4-3.7]	3.7 [3.5-3.8]	3.8 [3.7-3.8]	3.6 [3.5-3.7]		3.5 [3.4-3.7]	3.7 [3.5-3.8]	3.8 [3.8-4]	3.7 [3.6-4]	3.5 - 4.7
**Calcium (mmol/L)**	2.27 [2.24-2.29]	2.02[#] [1.99-2.09]	2.16[#] [2.15-2.21]	2.15[#] [2.13-2.16]	2.12[#] [2.09-2.15]		2.16[#] [2.14-2.21]	2.18[#] [2.13-2.21]	2.35 [2.3-2.38]	2.34 [2.32-2.38]	2.20 - 2.65
**Calcium corrected for albumin (mmol/L)**	2.33 [2.31-2.36]	2.21 [2.2-2.23]	2.3 [2.29-2.31]	2.29 [2.28-2.29]	2.26 [2.24-2.27]		2.35 [2.33-2.36]	2.33 [2.32-2.35]	2.36 [2.34-2.41]	2.37 [2.34-2.39]	2.20 - 2.65
**Phosphate (mmol/L)**	0.88 [0.8-0.96]	0.64[#] [0.55-0.69]	0.58[#] [0.5-0.73]	0.78[#] [0.67-0.88]	0.92 [0.9-0.96]		0.93 [0.88-1.01]	0.94 [0.86-0.98]	0.89 [0.84-0.96]	1.04 [0.88-1.09]	0.80 - 1.40
**Magnesium (mmol/L)**	0.75 [0.74-0.75]	0.68[#] [0.65-0.7]	0.74 [0.73-0.77]	0.74 [0.73-0.74]	0.72 [0.71-0.73]		0.75 [0.74-0.77]	0.74 [0.73-0.77]	0.76 [0.74-0.77]	0.78 [0.77-0.8]	0.70 - 1.10
**Urea (mmol/L)**	4.2 [3.2-5.1]	3.8 [3.1-4.6]	3.5 [2.7-4.4]	3.2 [2.6-4.1]	3 [2.3-4]		3.1 [2.2-4.2]	3.2 [2.4-4.2]	2.9 [2.6-3.7]	4.3 [4.1-4.8]	2.5 - 7.0
**Creatinine (umol/L)**	64 [61-66]	59[#] [56-60]	58[#] [56-62]	56[#] [56-58]	55[#] [54-57]		58[#] [56-60]	58[#] [57-61]	66 [64-69]	71 [69-72]	60 - 110
**Glomerular filtration rate (ml/min/1.73m^2)**	90 [90-90]	90 [90-90]	90 [90-90]	90 [90-90]	90 [90-90]		90 [90-90]	90 [90-90]	90 [90-90]	90 [90-90]	>90
**ALAT (U/L)**	19 [17-23]	15 [14-20]	16 [14-21]	14 [13-20]	15 [13-19]		15 [14-19]	14 [13-19]	17 [14-24]	18 [16-29]	<45
**ASAT (U/L)**	20 [19-24]	19 [16-22]	20 [16-23]	18 [17-22]	19 [18-20]		19 [16-24]	17 [15-22]	22 [18-27]	22 [19-25]	<35
**LDH (U/L)**	157 [151-162]	148 [141-153]	145 [138-154]	147 [146-151]	150 [147-155]		155 [147-164]	154 [153-161]	171 [168-175]	169 [158-187]	<250
**Creatine kinase (U/L)**	92 [70-97]	79 [63-86]	84 [67-94]	84 [67-88]	80 [64-85]		78 [61-88]	78 [60-83]	86 [82-87]	92 [74-105]	<170

**Alkaline phosphatase (U/L)**	52 [49-58]	46 [44-53]	50 [46-54]	48 [46-54]	50 [46-53]		52 [48-55]	51 [48-54]	58 [55-62]	56 [52-65]	<115
**Albumin (g/L)**	38 [37-38]	34$^{\#}$ [32-35]	35 [34-36]	35 [34-35]	34$^{\#}$ [33-35]		34$^{\#}$ [32-35]	34$^{\#}$ [32-35]	39 [37-42]	40 [39-41]	35 - 50
**C-reactive protein (mg/L)**	1 [1-1]	1 [1-1]	1 [1-1]	1 [1-1]	1 [1-1]		1 [1-1]	1 [1-1]	1 [1-2]	1 [1-1]	<10
**Iron (umol/L)**	19 [15-23]	18 [14-20]	21 [18-22]	23 [20-26]	28 [26-34]		36$^{\#}$ [28-42]	38$^{\#}$ [29-43]	15 [15-16]	20 [15-22]	10 - 30
**Total iron binding capacity (umol/L)**	68 [62-70]	61 [57-65]	66 [62-71]	62 [60-68]	64 [60-67]		64 [62-68]	64 [60-71]	74 [68-80]	76 [70-80]	45-80
**Chromium (mcg/L)**			0.6 [0.56-0.61]	2.62$^{\#}$ [1.72-2.75]			3.93$^{\#}$ [2.72-4.52]		2.86$^{\#}$ [1.55-2.95]	1.35$^{\#}$ [1.07-1.68]	0.38 – 0.90
**Manganese (mcg/L)**			0.63 [0.58-0.73]	0.96 [0.86-1.13]			0.88 [0.82-0.94]		0.53 [0.52-0.58]	0.55 [0.5-0.62]	0.35 – 1.10

All laboratory parameters are presented as median with interquartile range.
$^{\#}$ = Values marked are outside of the normal range. nr = no reference range available; MCV = mean corpuscular volume; MCH = mean cell haemoglobin; MCHC = mean cell haemoglobin concentration; RCDW = red cell distribution width; APTT = activated partial thromboplastin time; ASAT = aspartate aminotransferase; ALAT = alanine aminotransferase; GGT = gamma-glutamyltransferase; LDH = lactate dehydrogenase.

Supplementary Table S8 – Cytokine concentrations of the healthy volunteers in the first-in-human study.

	Baseline	0 hours	1 hour	1.5 hours	2 hours	2.5 hours	3 hours	3.5 hours	4 hours	5 hours	6 hours	8 hours	Day 1	Day 7	Reference range
	Heparin infusion														
			Extracorporeal circulation												
**TNF (pg/mL)**		6.23 [5.43-7.04]	6.04 [5.7-6.32]	6.91 [5.73-8.47]	6.61 [6.27-8.23]	5.92 [5.71-6.43]	6.63 [5.89-8.75]	6.3 [5.88-9.54]	6.69 [6.42-9.06]	6.42 [6.16-6.98]	6.08 [5.93-6.34]	6.23 [5.06-6.72]	5.66 [4.82-6]	5.32 [5.32-5.44]	nr
**IL-6 (pg/mL)**		6.46 [3.43-10.6]	7.02 [3.4-12.12]	7.5 [3.69-13.48]	7.2 [3.58-12.42]	7.68 [3.73-12.69]	6.05 [3.35-13.36]	8.04 [3.63-12.81]	8.18 [3.66-13.78]	9.52 [4.27-13.38]	8.33 [3.7-12.44]	6.41 [3.38-11.68]	6.25 [3.45-11.56]	5.68 [3.2-11.87]	nr
**IL-8 (pg/mL)**		12.21 [5.43-16.41]	13.7 [5.88-18.36]	14.62 [6.61-17.84]	13.68 [6.22-17.75]	13.63 [6.04-17.51]	12.87 [6.55-18.01]	13.03 [8.43-18.9]	14.52 [8.22-20.8]	16.03 [8.84-21.82]	15.57 [8.48-20.73]	15.3 [7.36-17.9]	13.65 [6.84-16.56]	11.37 [4.98-16.98]	nr
**IL-10 (pg/mL)**		4.3 [3.2-7.15]	4.18 [3.2-5.33]	4.06 [3.2-5.45]	4.39 [3.25-5.91]	4.38 [3.3-5.5]	4.82 [3.52-5.68]	5.28 [3.69-6.09]	4.38 [3.3-6.2]	4.41 [3.2-5.79]	4.06 [3.2-5.1]	3.84 [3.2-5.6]	3.73 [3.2-5.11]	3.73 [3.2-4.94]	nr
**IL-1RA (pg/mL)**		10.22 [9.26-14.53]	12.91 [9.75-14.35]	15.69 [14.43-17.62]	15.51 [10.05-19.36]	14.7 [9.68-15.63]	13.41 [10.71-15.38]	13.01 [8.13-14.89]	15.84 [8.8-17.87]	13.86 [10.22-16.88]	14.02 [10.77-15.63]	10.88 [8.9-14.86]	10.4 [7.6-14.12]	7.97 [5.8-9.66]	nr
**IP-10 (pg/mL)**		761 [704.75-792.5]	779 [671.25-848.5]	815.5 [664.75-977.5]	846 [692.75-943.75]	799.5 [665.5-903.5]	807.5 [623.5-907.5]	768.5 [648-929.5]	776.5 [668.5-968.5]	779.5 [648.5-1022.25]	647.5 [595.25-836.25]	505.5 [459.75-654.75]	351.5 [287.5-408]	217.5 [187.5-234]	nr
**GCSF (pg/mL)**		11.23 [9.11-21.6]	10.16 [6.1-24.06]	17.14 [14.16-21.03]	19.92 [8.25-26.79]	15.54 [7.16-20.94]	17.72 [14.45-21.03]	17.72 [14.45-20.94]	19.87 [11.26-22.1]	12.9 [9.94-16.63]	14.49 [10.17-20.5]	19.92 [13.76-22.13]	15.54 [10.17-18.55]	12.9 [5.51-16.63]	nr

**MCP1 (pg/mL)**		140 [136.5-157.75]	154.5 [146.5-164]	158.5 [153.25-166.75]	153 [140.5-158.75]	150.5 [143.25-169.75]	158 [137.5-165]	164.5 [160-169.75]	182 [158.5-192.75]	202 [169-229.75]	201 [181.25-251.5]	188 [167.25-214.75]	167 [153.75-187.75]	148 [128-153.75]	nr
**MIP1a (pg/mL)**		3.2 [3.2-6.18]	3.2 [3.2-6.85]	4.15 [3.35-4.79]	4.48 [3.25-5.57]	3.2 [3.2-5.23]	4.31 [3.25-8]	5.64 [3.2-8.39]	5.7 [3.53-8.41]	3.49 [3.2-8.04]	3.3 [3.2-7.54]	3.2 [3.2-5.23]	3.2 [3.2-3.91]	3.2 [3.2-3.2]	nr
**MIP1b (pg/mL)**		11.07 [10.32-11.4]	11.95 [10.98-12.49]	12.7 [11.88-13.71]	14.63 [13.41-16.42]	14.95 [13.77-17.53]	15.75 [15.15-18.92]	14.84 [13.23-22.71]	16.63 [14.99-21.98]	16.91 [15.39-20.74]	16.76 [14.25-22.03]	14.45 [12.01-17.42]	12.51 [11.22-13.79]	11.75 [10.54-13.33]	nr

All cytokine concentrations are presented as median with interquartile range.
TNF = tumour necrosis factor; nr = no reference range available; IL = interleukin; IL-1RA = interleukin-1 receptor antagonist; IP = interferon gamma induced protein; GCSF = granulocyte colony-stimulating factor; MCP = monocyte chemoattractant protein; MIP = macrophage inflammatory protein.

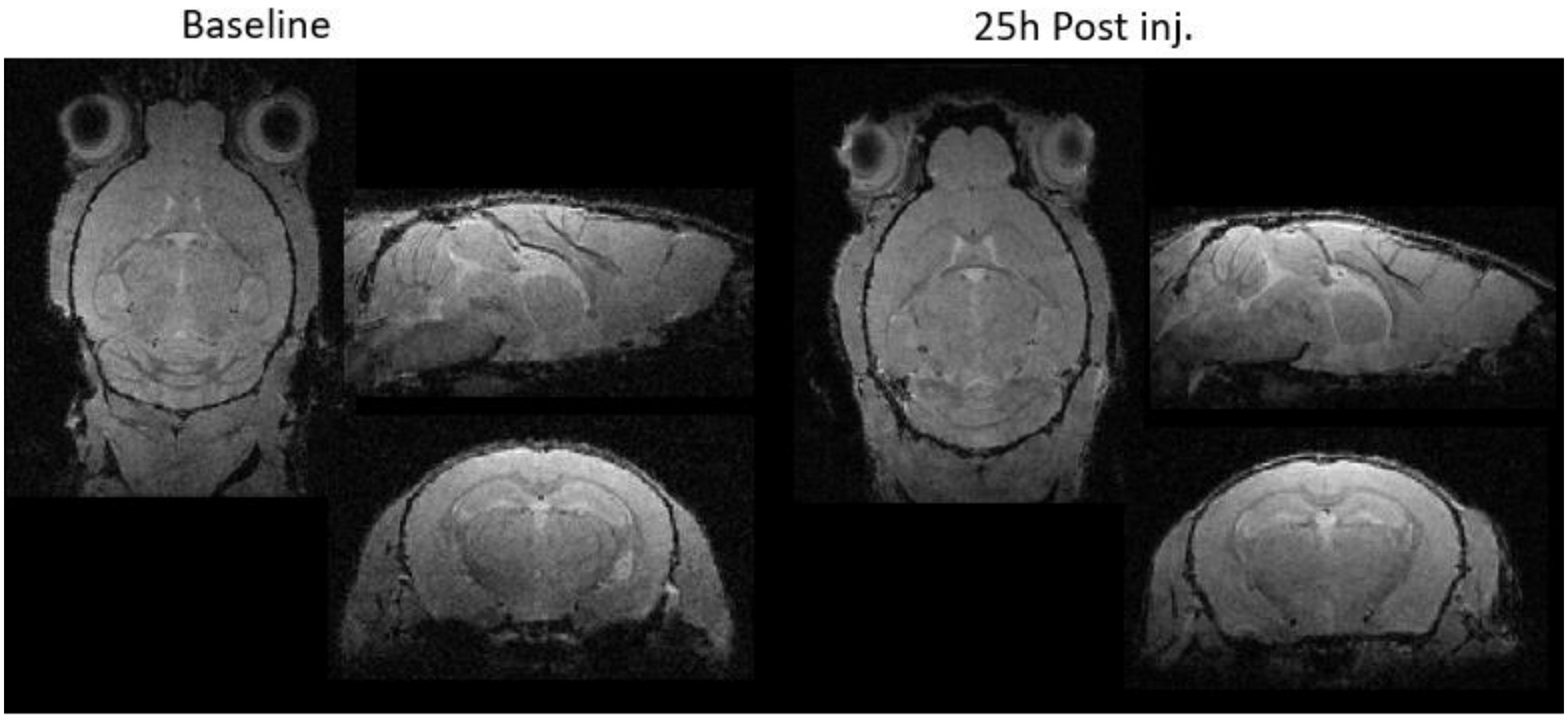

Supplementary Figure S1. Brain MRI of a representative mouse 25 hours after injection of magnetic beads in the biodistribution of Anti-IL-6 Beads study. High resolution T2* weighted 3D gradient echo images (FLASH) showed no parenchymal beads in radiological screening and no global or cortical signal decrease in regions of interest analysis 25 hours after Anti-IL-6 Bead injection as compared to baseline. MRI voxel size 100x100x100 μm^3.

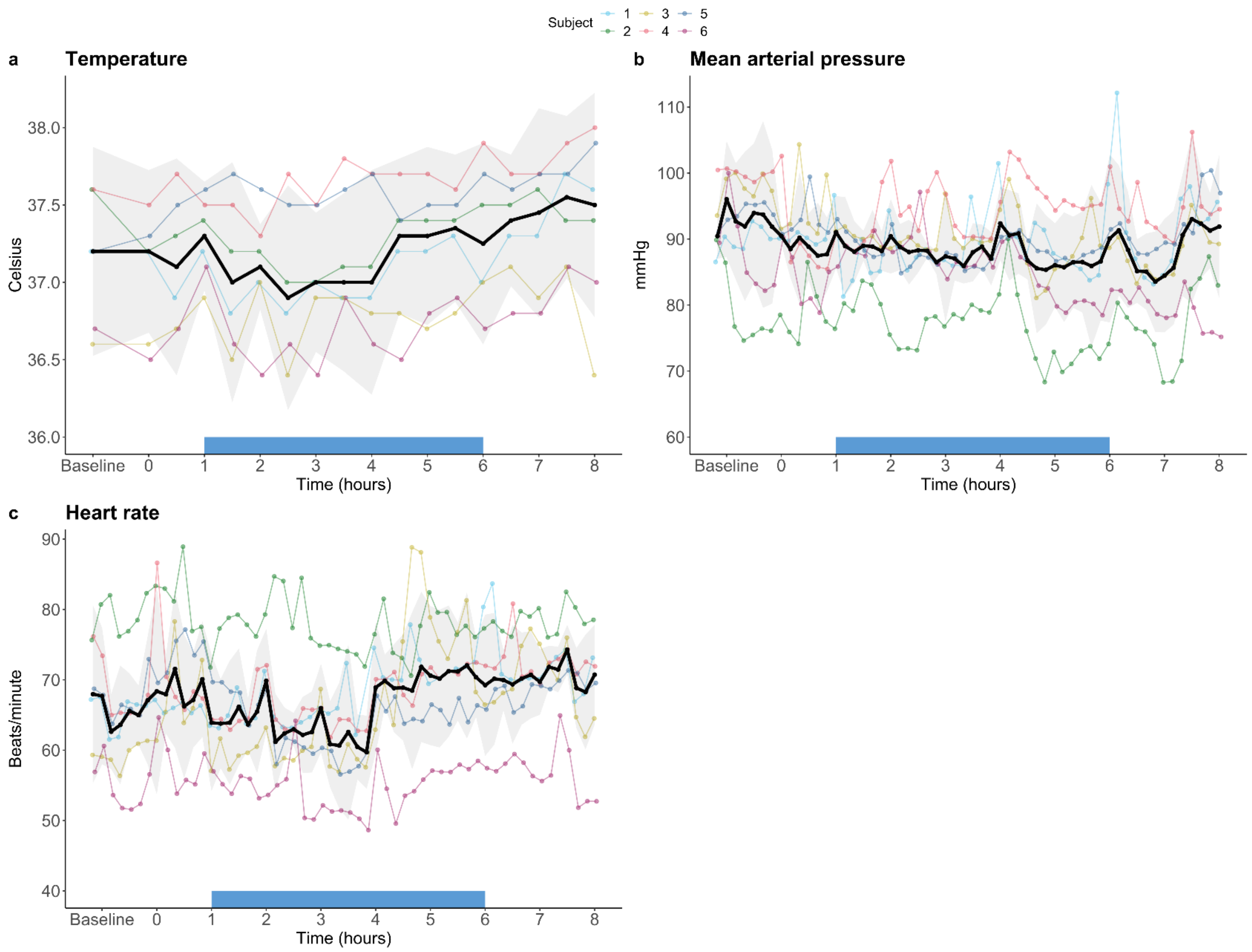

Supplementary Figure S2. Changes over time in vital signs during the experiment day of the first-in-human study. Data are displayed per individual subject (coloured lines) and as median ± IQR (black line with grey area). The extracorporeal circulation period is indicated by the blue bar on the x-axis.

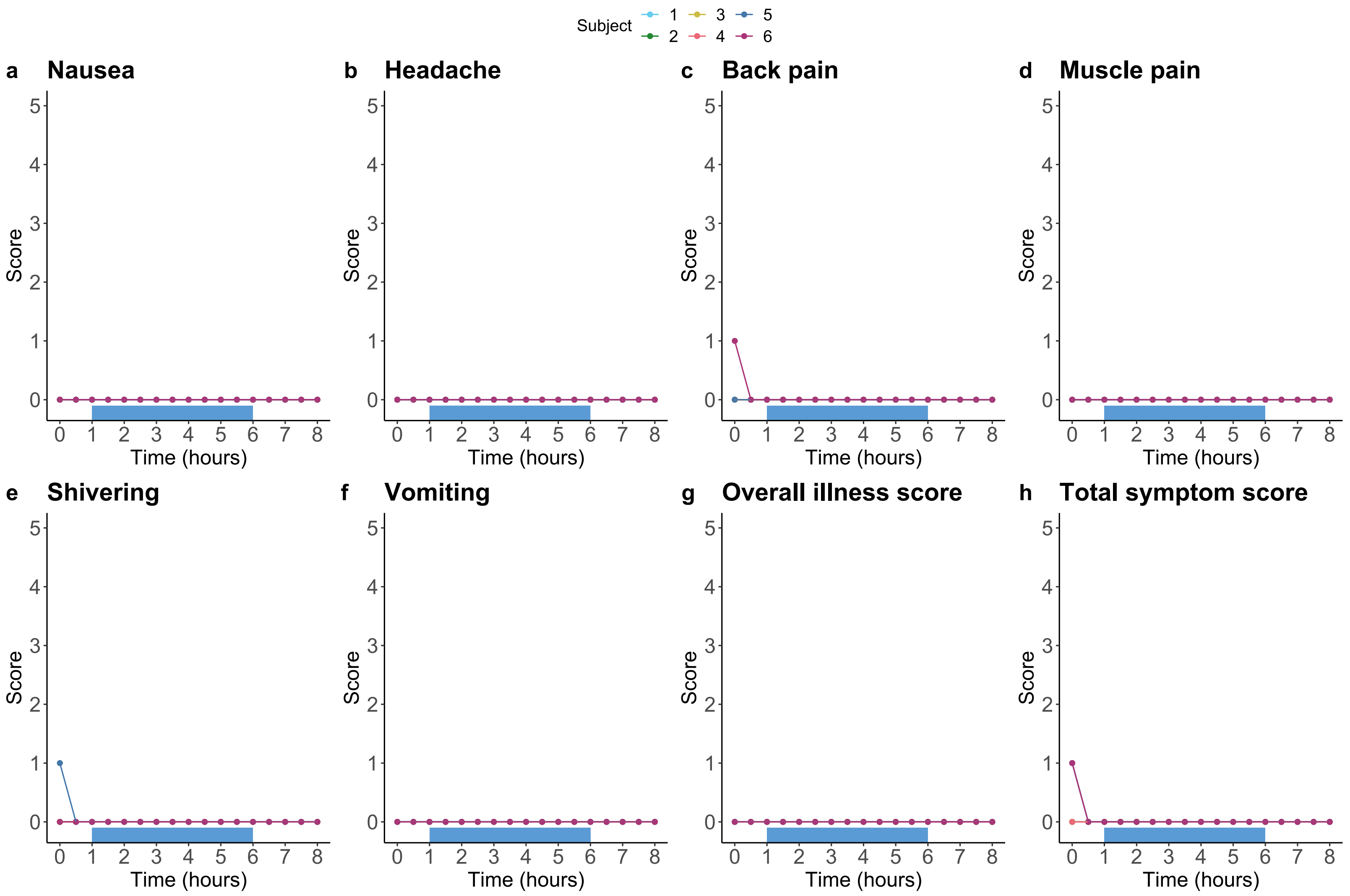

Supplementary Figure S3. Changes over time in symptoms during the experiment day of the first-in-human study. The total symptom score was calculated by the sum of all symptoms, including the overall illness score. Data are displayed per individual subject (coloured lines). The extracorporeal circulation period is indicated by the blue bar on the x-axis.
